# A subset of human dermal fibroblasts overexpressing Cockayne syndrome group B protein resist UVB radiation‐mediated premature senescence

**DOI:** 10.1111/acel.14422

**Published:** 2024-12-19

**Authors:** Asimina Fotopoulou, Maria T. Angelopoulou, Harris Pratsinis, Eleni Mavrogonatou, Dimitris Kletsas

**Affiliations:** ^1^ Laboratory of Cell Proliferation and Ageing, Institute of Biosciences and Applications National Centre for Scientific Research “Demokritos” Athens Greece; ^2^ Department of Chemistry University of Patras Patras Greece

**Keywords:** CSB, human dermal fibroblasts, photoaging, resistance, RNA‐seq, senescence, UVB

## Abstract

Ultraviolet B (UVB) radiation is a major contributor to skin photoaging. Although mainly absorbed by the epidermis, UVB photons managing to penetrate the upper dermis affect human dermal fibroblasts (HDFs), leading, among others, to the accumulation of senescent cells. In vitro studies have shown that repeated exposures to subcytotoxic UVB radiation doses provoke HDFs' premature senescence shortly after the end of the treatment period. Here, we found that repetitive exposures to non‐cytotoxic UVB radiation doses after several days lead to mixed cultures, containing both senescent cells and fibroblasts resisting senescence. “Resistant” fibroblasts were more resilient to a novel intense UVB radiation stimulus. RNA‐seq analysis revealed that ERCC6, encoding Cockayne syndrome group B (CSB) protein, is up‐regulated in resistant HDFs compared to young and senescent cells. CSB was found to be a key molecule conferring protection toward UVB‐induced cytotoxicity and senescence, as siRNA‐mediated CSB loss‐of‐expression rendered HDFs significantly more susceptible to a high UVB radiation dose, while cells from a CSB‐deficient patient were found to be more sensitive to UVB‐mediated toxicity, as well as senescence. UVB‐resistant HDFs remained normal (able to undergo replicative senescence) and non‐tumorigenic. Even though they formed a distinct population in‐between young and senescent cells, resistant HDFs retained numerous tissue‐impairing characteristics of the senescence‐associated secretory phenotype, including increased matrix metalloprotease activity and promotion of epidermoid tumor xenografts in immunodeficient mice. Collectively, here we describe a novel subpopulation of HDFs showing increased resistance to UVB‐mediated premature senescence while retaining undesirable traits that may negatively affect skin homeostasis.

## Graphical abstract

A pre‐existing subpopulation of human dermal fibroblasts (HDFs) overexpressing Cockayne syndrome group B (CSB) protein resist ultraviolet B (UVB) radiation‐induced premature senescence and coexist in mixed cultures with UVB‐induced senescent HDFs. UVB‐resistant HDFs remain normal and form a distinct population in‐between young and senescent cells, while retaining undesirable traits that may negatively affect skin homeostasis.

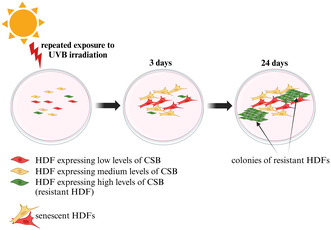

**Abbreviations**
ANOVA: analysis of variance
ATM: ataxia telangiectasia mutated
BrdU: 5‐bromo‐2′‐deoxyuridine
BSA: bovine serum albumin
CCCP: carbonyl cyanide m‐chlorophenyl hydrazone
CSB: Cockayne syndrome group B
DAPI: 4′,6‐diamidino‐2‐phenylindole
DDR: DNA damage response
DEGs: differentially expressed genes
DMEM: Dulbecco's modified Eagle medium
ECM: extracellular matrix
EGFR: epidermal growth factor receptor
FBS: fetal bovine serum
FITC: fluorescein isothiocyanate
GAPDH: glyceraldehyde‐3‐phosphate dehydrogenase
GO: gene ontology
HDF: human dermal fibroblast
HIF‐1: hypoxia‐inducible factor‐1
HRP: horseradish peroxidase
ICAM‐1: intercellular adhesion molecule 1
IGFBP: insulin‐like growth factor‐binding protein
IL: interleukin
iPSCs: induced pluripotent stem cells
JNKs: c‐Jun N‐terminal kinases
LD_50_
: lethal dose 50
Mdm2: mouse double minute 2
MDS: multidimensional scaling
MIP: maximum intensity projection
MMP: matrix metalloprotease
MSCs: mesenchymal stem cells
Nrf2: nuclear factor erythroid 2‐related factor 2
PBS: phosphate‐buffered saline
PUVA: 8‐methoxypsoralen followed by UVA irradiation
PVDF: polyvinylidene fluoride
RNA‐seq: RNA sequencing
ROS: reactive oxygen species
RT‐qPCR: reverse transcription quantitative polymerase chain reaction
SASP: senescence‐associated secretory phenotype
SCID: severe combined immunodeficiency
SDS‐PAGE: sodium dodecyl‐sulfate polyacrylamide gel electrophoresis
SIPS: stress‐induced premature senescence
siRNA: small interfering RNA
TCR: transcription‐coupled DNA repair
TGFβ: transforming growth factor beta
UV: ultraviolet
UVA: ultraviolet A
UVB: ultraviolet B
α‐SMA: alpha smooth muscle actin

## 1 INTRODUCTION

The skin is the outermost layer of the human body, constantly confronting any external environmental threat, thus serving as a protective barrier for internal organs. Hence, cutaneous aging—the most apparent and direct manifestation of the organismal aging process—is the combined outcome of intrinsic (the result of chronological age affecting all body organs) and extrinsic (the response to environmental insults) aging (Gilchrest, 2013; Sanches Silveira & Myaki Pedroso, 2014; Toutfaire et al., 2017). Among the most important extrinsic aging factors is solar ultraviolet (UV) irradiation, which can be harmful for the skin, causing from short‐term, transient, and repairable dermatitis and sunburn to long‐term photoaging and even photocarcinogenesis (Bosch et al., 2015; Gilchrest, 2013; Sanches Silveira & Myaki Pedroso, 2014; Toutfaire et al., 2017). Both the epidermis and the dermis are affected by sunlight; however, major structural and functional alterations of the photo‐aged skin, for example, wrinkles' formation, pigmentation, rigidity, and loss of elasticity due to aberrant collagen and elastin synthesis and degradation (Bosch et al., 2015; Gilchrest, 2013; Huertas et al., 2016), are mostly associated with disturbed homeostasis of the dermis and of fibroblasts therein, the latter being its main cellular components and extracellular matrix (ECM) producers (Pittayapruek et al., 2016; Quan et al., 2004; Wlaschek et al., 2021).

The most detrimental component of the terrestrial UV irradiation is its shorter UVB waveband (280–315 nm), as it delivers higher energy levels than UVA (315–400 nm) (Gilchrest, 2013). Even though mostly absorbed in the epidermis, 5%–10% of UVB photons penetrate and reach the papillary dermis, exerting their genotoxic effects in the embedded fibroblasts (Gilchrest, 2013; Sanches Silveira & Myaki Pedroso, 2014), by directly interacting with their DNA or by generating reactive oxygen species (ROS) (Cadet et al., 2012, 2015; Svobodová et al., 2012). It has been reported that acute and chronic exposure to UVB radiation leads to the selective loss of fibroblasts from the upper dermis of the human and mouse skin (Mavrogonatou et al., 2022; Rognoni et al., 2021). We have recently shown that human dermal fibroblasts (HDFs) cope with acute cytotoxic UVB doses by activating two biochemical pathways functioning synergistically, the indispensable JNKs/ATM‐p53 axis and the auxiliary EGFR/Akt and Nrf2 pathways (Mavrogonatou et al., 2022).

In addition, accumulation of senescent cells has long been observed in aged and sun‐exposed skin (Demaria et al., 2015; Ressler et al., 2006; Waaijer et al., 2012). Cellular senescence is characterized by growth arrest, reached after a limited number of cell divisions due to the progressive loss of telomere length (replicative senescence), while it could also be a response to various extrinsic genotoxic stimuli, including oxidative stress, oncogenic activation, exposure to chemotherapeutic drugs, and ionizing or ultraviolet (UV) radiation (stress‐induced premature senescence, SIPS) (Gorgoulis et al., 2019). Senescent cells express a distinctive pro‐inflammatory and catabolic phenotype, called the “senescence‐associated secretory phenotype” (SASP) affecting tissue homeostasis (Coppé et al., 2008; Gorgoulis et al., 2019; Mavrogonatou et al., 2023). HDFs driven to premature senescence after being repeatedly exposed to mild, subcytotoxic, or non‐cytotoxic doses of UVB radiation in vitro have served as an established experimental model to study the mechanisms underlying cutaneous photoaging (Borlon et al., 2008; Cavinato et al., 2017; Zeng et al., 2014). These cells have been characterized, among others, by increased matrix metalloproteases (MMPs)’ secretion and decreased collagen production contributing to aged skin (Campisi, 1998; Permatasari et al., 2014; Wlaschek et al., 2021).

In the existing literature, several protocols for the induction of UVB‐mediated SIPS have been reported, using various doses of UVB radiation, different numbers of exposures, etc. Interestingly, in all of them, as far as we know, the induction of senescence has been assessed only a few days following the last irradiation dose. Having in mind that a full senescent phenotype induced by excessive genotoxic stimuli (such as ionizing radiation, genotoxic drugs, oxidative stress, etc.) in human fibroblasts requires a much longer period to be expressed, we repeatedly exposed HDFs to non‐cytotoxic UVB radiation doses and extended the time frame of observation to monitor the long‐term effect of this treatment on the cells. Thereby we identified and characterized a population of HDFs showing resistance to UVB‐mediated SIPS and possessing a unique molecular profile with putative effect on skin homeostasis.

## 2 MATERIALS AND METHODS

### 2.1 Cells and cell culture conditions

Primary HDFs from two different donors used in this study were from the pre‐existing cell bank of the Laboratory of Cell Proliferation and Ageing (Armatas et al., 2014; Mavrogonatou et al., 2022). Neonatal foreskin fibroblasts (AG01523) were supplied from the Coriell Institute for Medical Research (Camden, NJ, USA). Foreskin fibroblasts FF95 were generously provided by Prof. K. Scharffetter‐Kochanek. Dermal fibroblasts from a Cockayne syndrome group B (CSB)‐deficient patient (GM00739) with a mutation in the ERCC6 gene resulting in the loss of a functional CSB protein, but normally expressing the fusion protein (Newman et al., 2008) were also provided by the Coriell Institute for Medical Research. The human skin carcinoma cell line A431 was purchased from the American Type Culture Collection (ATCC, Rockville, MD, USA). All cells were routinely cultured in Dulbecco's Modified Eagle Medium (DMEM) supplemented with penicillin (100 U/mL)/streptomycin (100 mg/mL) (obtained from Biosera, Nuaillé, France) and 10% (v/v) FBS (from Gibco BRL, Invitrogen, Paisley, UK), were maintained in a humidified atmosphere of 5% CO_2_ at 37°C, and were subcultured when confluent using a trypsin/citrate (0.25%:0.30% w/v) solution. Cells were regularly tested to ensure their mycoplasma‐free status.

### 2.2 γ‐Irradiation‐induced premature senescence

For the induction of premature senescence, primary early‐passage (young) HDFs were treated with γ‐irradiation in a ^60^Co gamma source (Gamma Chamber 4000A, Isotope Group, Bhadha, Atomic Research Company, Trombay, Bombay, India), as previously described (Kwiatkowska et al., 2023; Liakou et al., 2016; Mavrogonatou et al., 2021; Papadopoulou & Kletsas, 2011). Cells were then subcultured until cease of their proliferation, as estimated by 5‐bromo‐2′‐deoxyuridine (BrdU) incorporation. Establishment of the ionizing radiation‐induced senescent (IS) phenotype was validated by multiple markers, including enhanced p16^INK4a^ protein levels, lamin B1 down‐regulation, negative nuclear Ki67 staining, and positive SenTraGor staining, in accordance with a recently described algorithmic assessment of cellular senescence (Kohli et al., 2021).

### 2.3 Treatment with UVB radiation

For UVB irradiation, confluent culture dishes were placed in the center of a UV box, as reported previously (Mavrogonatou et al., 2022), under a UVB lamp (Sankyo Denki Co., Kanagawa, Japan) with an emission spectrum of 280–360 nm and a peak at 306 nm, after aspiration of the culture medium and its replacement by a thin layer of phosphate‐buffered saline (PBS). Right after UVB irradiation, PBS was replaced by fresh culture medium supplemented with 10% (v/v) FBS, and cells were further incubated at 37°C for the selected time periods according to the experimental design. The intensity of the applied UVB light in mW/cm^2^ was measured using chemical actinometry, based on the photolysis of a potassium ferrioxalate solution (Hatchard & Parker, 1956). Total energy of the irradiation dose hitting the sample in mJ/cm^2^ was calculated by multiplying the intensity value with the duration of exposure in sec. Dermal fibroblasts were exposed to a single dose of UVB radiation (from 12 to 3150 mJ/cm^2^) or to repeated doses of 35, 50, or 70 mJ/cm^2^ twice per day for five consecutive days. Cells not exposed to the UVB lamp served as the untreated control.

### 2.4 Estimation of cell viability

For the estimation of cell viability, cells were incubated at 37°C for 72 h post‐treatment. At the end of the incubation period, cells were detached by trypsinization, resuspended in culture medium, and stained with the vital dye neutral red (Sigma, St. Louis, MO, USA) at a final concentration of 80 μg/mL. After a 15‐min incubation with the staining solution at 37°C, red‐colored metabolically active cells were measured using a hemocytometer (Bourkoula et al., 2018; Mavrogonatou et al., 2022).

Alternatively, the neutral red uptake assay performed in 96‐well tissue culture plates was used (Repetto et al., 2008) with slight modifications. In brief, HDFs were plated in 96‐well flat, clear‐bottom black, polystyrene, tissue culture‐treated microplates in serum‐containing culture medium. After treatment, culture medium was aspirated and replaced by neutral red dissolved in serum‐free, phenol‐red‐free DMEM at a final concentration of 20 μg/mL for a further 4‐h incubation. Then, the cells were washed once with PBS, and neutral red was extracted in each well using an acidified ethanol solution. Fluorescence was measured by a Tecan Spark microplate reader (Tecan Group Ltd., Maennedorf, Switzerland) with excitation and emission wavelengths of 530 and 645 nm, respectively. Cell viability was expressed as a % ratio of untreated cells.

### 2.5 Assessment of cell viability as a response to various stresses

In order to evaluate the ability of the three different populations of HDFs (young, resistant, and IS cells) to tolerate different kinds of stressful stimuli, neutral red was used as described above. To assess the response of the cells to oxidative stress (after treatment with 0–1000 μΜ H_2_O_2_ and 0–500 μΜ NaAsO_2_), mitochondrial stress (induced by carbonyl cyanide m‐chlorophenyl hydrazone, CCCP, at concentrations of 0–800 μΜ) and direct genotoxic stress (after exposure to 0–25 μΜ doxorubicin) 72 h post‐treatment, the neutral red uptake assay was performed. Especially in the case of exposure to heat stress (43°C for 3, 6, and 9 h) or to a single dose of UVB radiation (560 mJ/cm^2^), cell counting in a hemocytometer after staining of the cells with neutral red was performed.

### 2.6 Immunofluorescence experiments

For the estimation of cell proliferation, cells were allowed to attach onto glass coverslips before labeling with 50 μM BrdU for 48 h, as previously described (Mavrogonatou et al., 2021; Papadopoulou et al., 2022). After fixation with 4% (v/v) formaldehyde in PBS for 10 min, permeabilization with 0.2% (v/v) Triton X‐100 in PBS for 10 min, denaturation of the DNA with 2 N HCl for 30 min, and blocking with 0.5% (v/v) gelatin for 30 min, samples were incubated overnight with an anti‐BrdU‐FITC antibody purchased from BioLegend (SanDiego, CA, USA) at 4°C. Counterstaining was performed with 4 μg/mL 4′,6‐diamidino‐2‐phenylindole (DAPI) dihydrochloride in PBS for 15 min at room temperature.

Immunofluorescence analysis for the estimation of CSB expression was performed as previously described (Batenburg et al., 2015; Horibata et al., 2004) with slight modifications. In brief, cells seeded on coverslips were fixed with 4% (v/v) formaldehyde in PBS for 10 min, washed thrice with PBS, and permeabilized with 0.5% (v/v) Triton X‐100 in PBS for 5 min. After blocking with 1% (w/v) bovine serum albumin (BSA) for 30 min, samples were incubated with an anti‐CSB antibody (Abcam, Cambridge, UK) for 3 h, washed thrice with PBS, incubated with a CF®488A‐conjugated anti‐mouse antibody (Biotium Inc., Fremont, CA, USA), and counterstained with 0.5 μg/mL DAPI in PBS for 15 min at room temperature.

Coverslips were mounted with 1 mg/mL p‐phenylenediamine in glycerol/PBS (9:1) on microscopy slides and observed under an upright Zeiss Axioplan 2 fluorescent microscope (Zeiss, Jena, Germany) and a confocal laser scanning microscope (TCS SP8 multiphoton confocal microscope, Leica, Mannheim, Germany).

The percentage of the cells with the ability to proliferate in a given population was calculated by dividing the number of BrdU‐positive nuclei by the number of the DAPI‐positive nuclei. Quantification of the distribution of the mean CSB fluorescence intensity for all nuclei in a given population of HDFs was performed in multichannel Z‐stack images captured using confocal laser scanning microscopy with the following workflow: Total pixel intensity in the corresponding area of the CSB channel maximum intensity projection (MIP) was calculated for each nucleus. To account for variations in nuclear size, total CSB fluorescence intensity per nucleus was normalized by the nucleus area, which was calculated from the segmented DAPI mask after processing of acquired DAPI‐stained images using a custom Python script with the *scikit‐image* and *NumPy* libraries. The presence of different subgroups within the young untreated HDF population was assessed via analysis of CSB mean fluorescence intensity distribution across the nuclei using the K‐means clustering algorithm, while the classification of the resistant population was performed based on the clustering of young cells. The *mean CSB fluorescence intensity* feature served as the input variable for clustering, while the optimal number of clusters *K* = 3 was determined based on exploratory analysis and visual inspection of the data.

### 2.7 Immunocytochemistry

SenTraGor and Ki67 staining procedures were performed as previously reported (Evangelou et al., 2017; Kolbe et al., 2021; Veroutis et al., 2021) with slight modifications. In brief, HDFs attached to glass coverslips were fixed with 4% (v/v) formaldehyde in PBS, membrane‐permeabilized with 0.3% Triton X‐100 in PBS, and incubated with peroxidase and protein block contained in the Novolink Polymer Detection System (Leica Biosystems, Newcastle, UK). Lipofuscin‐containing cells were stained with the SenTraGor reagent (Lab Supplies Scientific, Athens, Greece), following the manufacturer's instructions. In brief, coverslips were subjected to one wash with PBS, followed by two washes with 50% (v/v) and 70% (v/v) ethanol. Coverslips were then incubated with the SenTraGor reagent at 37°C in the dark until detection of a light brown signal under a light microscope. After removing the excess of SenTraGor stain with 50% (v/v) ethanol, samples were incubated with an anti‐biotin antibody (Abcam) overnight at 4°C. For the staining of the cellular marker of proliferation Ki67, coverslips were incubated with an anti‐Ki67 antibody (Cell Marque, Rocklin, CA, USA) overnight at 4°C. Development of the signal was achieved using the DAB Chromogen diluted in the DAB Substrate Buffer contained in the Novolink Polymer Detection System (Leica Biosystems), according to the manufacturer's instructions. Hematoxylin was used for counterstaining.

### 2.8 Colony formation assay

The colony formation assay was performed to assess the ability of single cells to grow into colonies (Franken et al., 2006). In brief, 1000 cells were plated onto the surface of a 100‐mm petri dish and incubated at 37°C, 5% CO_2_ for 10, 17, and 24 days. Culture medium was changed into a fresh one every 7 days. After fixation with 4% (v/v) formaldehyde in PBS for 30 min, samples were stained with 0.01% (w/v) crystal violet in double‐distilled (dd) H_2_O for 1 h at room temperature. At the end of the incubation time with the staining solution, excess of the dye was washed with ddH_2_O, and colonies were observed and photographed under an ECLIPSE Ts2 inverted microscope (Nikon, Tokyo, Japan) using a Basler microscopy camera and software for image acquisition (Basler, Ahrensburg, Germany).

### 2.9 RNA sequencing (RNA‐seq)

Total RNA was extracted with Trizol reagent (Invitrogen), and RNA concentration and purity were determined using a Nanodrop ND‐1000 spectrophotometer (Nanodrop Technologies, Wilmington, DE, USA). RNA quality was determined on an Agilent 2100 Bioanalyzer (Agilent Technologies, Santa Clara, CA, USA) using the Agilent RNA 6000 Nano Kit reagents and protocol. For library preparation, the 3′ mRNA‐Seq Library Prep Kit Protocol for Ion Torrent (QuantSeq‐LEXOGEN™, Vienna, Austria) was used according to the manufacturer's instructions. Briefly, up to 500 ng of RNA from each sample were used for first strand synthesis, after which remaining RNA was removed and second strand synthesis was initiated by a random primer containing Ion Torrent‐compatible linker sequences at its 5′ end. In‐line barcodes were introduced, and second‐strand synthesis was followed by magnetic bead‐based purification; the resulting libraries were amplified for up to 15 cycles and repurified. Quality and quantity of libraries were assessed on a bioanalyzer using the DNA High Sensitivity Kit reagents and protocol (Agilent Technologies). The quantified libraries were pooled at a final concentration of 7 pM, templated and enriched on the Ion Proton One Touch system using the Ion PI™ Hi‐Q™ OT2 200 Kit (Thermo Fisher Scientific, Cleveland, OH, USA) and sequenced using the Ion PI™ Hi‐Q™ Sequencing 200 Kit on Ion Proton PI™ V2 chips (Thermo Fisher Scientific) on an Ion Proton™ System, according to the manufacturer's instructions.

FASTQ files were trimmed with Trim Galore (v.0.6.51) to remove low‐quality read ends using a Phred score of 20. Subsequently, a two‐step alignment procedure was applied. Pre‐processed reads were aligned against the hg38 reference genome (Ensembl) with HISAT2 (v.2.2.1), and then the reads left unmapped were subjected to a second alignment round using BOWTIE2 (v.2.3.5.1) with the local and very‐sensitive local switches turned on.

Downstream analysis of the resulting BAM files was performed with the *metaseqR* ^*2*^ R package (v.1.9.2) (Fanidis & Moulos, 2021). Briefly, the raw BAM files, one per sample, were summarized to a 3′ UTR reads count table using the package *GenomicRanges* (v1.44.0) (Lawrence et al., 2013) and Ensembl human genome hg38. For the UTR counting, the entire 3′ UTR region, with a minimum length of 300 base pairs and 50 base pairs to flank the UTR end, was taken into consideration. In the resulting reads count table, each row represented one 3′ UTR region and each column a Quant‐seq sample. Next, reads were summarized per gene, and the returned gene count table was normalized using the Bioconductor package *DESeq* after removing genes having zero reads across all samples (Anders & Huber, 2010). Post‐normalization, gene counts were filtered for possible artifacts using default gene filtering options.

### 2.10 Differential gene expression and functional gene set analysis

The filtered gene counts table was subjected to differential expression analysis for the contrasts resistant versus young cells, IS versus young cells, and IS versus resistant cells using sequentially *DESeq*, *edgeR*, *limma*, *NBPSeq*, and *NOISeq* methods supported by *metaseqR* ^*2*^. Their *p*‐values were then combined by the PANDORA algorithm to account, among others, for the false positives reported (Fanidis & Moulos, 2021; Moulos & Hatzis, 2015). Benjamini‐Hochberg‐corrected PANDORA *p*‐values of less than 0.05 and absolute fold change of at least 1.2 were used as differential expression thresholds.

Functional annotation of the up‐regulated and down‐regulated between selected experimental groups gene lists was performed with ConsensusPathDB (Kamburov et al., 2009, 2011) for all three gene ontology (GO) categories (biological process, molecular function, and cellular compartment), ontology level 2, and *p*‐value cutoff 0.01. The GO term *response to stress* was selected from the resulting enriched GO‐based sets for further analysis.

### 2.11 Reverse transcription quantitative polymerase chain reaction (RT‐qPCR)

Gene expression analysis was performed by reverse transcription quantitative polymerase chain reaction (RT‐qPCR), as described previously (Mavrogonatou et al., 2021, 2022). cDNA was synthesized from 500 ng of total RNA in 10‐μL reactions using the PrimeScript RT Reagent Kit (Takara, Tokyo, Japan), according to the manufacturer's instructions. 20‐μL qPCR reactions were performed with cDNA at a final dilution of 1:100 using the KAPA SYBR FAST qPCR kit (KAPA Biosystems, Boston, MA, USA) in a CFX96 in vitro diagnostics real‐time PCR system accompanied by the CFX Manager software v. 2.1 (Bio‐Rad, Hercules, CA, USA). mRNA levels were quantified in comparison to the untreated control using the $2^{-\Delta \Delta C_{T}}$ method (Livak & Schmittgen, 2001) and glyceraldehyde‐3‐phosphate dehydrogenase (GAPDH) as the reference gene. Sequences of the primers used in the current study are presented in Table 1.

**TABLE 1:** Primer sequences (5′ → 3′).

Target gene	Forward primer	Reverse primer
*p21* ^*WAF1*^	CTGGAGACTCTCAGGGTCGAA	CCAGGACTGCAGGGTTCCT
*icam1*	TATGGCAACGACTCCTTCT	CATTCAGCGTCACCTTGG
*il6*	CTTTTGGAGTTTGAGGTATACCTAG	CGCAGAATGAGATGAGTTGTC
*il8*	TGCCAAGGAGTGCTAAAG	CTCCACAACCCTCTGCAC
*col1a*	CCAGAAGAACTGGTACATCA	CCGCCATACTCGAACTGGAA
*fibronectin*	CTGAAGAATAATCAGAAGAGC	ACCATGTTCCTCAAAGATC
*acta2*	TCTATGCCTCTGGACGCACAAC	TCTCACGCTCAGCAGTAGTAACG
*tgfβ1*	TGCCACAGATCCCCTATTCAAG	AGGGAGAAGGGCGCAGTG
*mmp1*	CCTTCTACCCGGAAGTTGAG	TCCGTGTAGCACATTCTGTC
*mmp2*	AAGAACCAGATCACATACAGGATCA	GTATCCATCGCCATGCTCC
*mmp3*	TTTTGGCCATCTCTCCTTCA	TGTGGATGCCTCTTGGGTATC
*mmp13*	TTGAGCTGGACTCATTGTCG	GGAGCCTCTCAGTCATGGAG
*serpinb2*	GGGTCAAGACTCAAACCAAAG	CCTTTGAAGTAGACAGCATTC
*serpine2*	TGGTGATGAGATACGGCGTAA	GTTAGCCACTGTCACAATGTCTT
*ctsk*	CCGCAGTAATGACACCCTTT	GCACCCACAGAGCTAAAAGC
*ctsb*	GGAGGGAGCTTTCTCTGTGT	CAGTAGGGTGTGCCATTCTC
*dcn*	CCTGATGACCGCGACTTCGAG	TTTGGCACTTTGTCCAGACCC
*igfbp5*	GGGTTTGCCTCAACGAAAAG	TTTCTGCGGTCCTTCTTCAC
*ercc6*	TCCTTCATCAACATCTCCAA	ATCTTCTGCTCTTCCACTAA
*gapdh*	GAGTCCACTGGCGTCTTC	GCATTGCTGATGATCTTGAGG

### 2.12 Western blot analysis

Total protein extraction and western blot analysis were performed as previously described (Mavrogonatou et al., 2022). In brief, HDFs' lysates were collected in hot Laemmli sample buffer supplemented with protease and phosphatase inhibitor cocktails (Sigma), boiled at 100°C for 3 min, sonicated for 15 s, clarified by centrifugation, and stored at −30°C until SDS‐PAGE. Separated proteins were then electro‐transferred from polyacrylamide gels to PVDF membranes (Thermo Scientific) and subjected to western blot analysis with primary antibodies raised against p16^INK4a^ (BD Pharmingen, San Diego, CA, USA), lamin B1 (Proteintech, Rosemont, IL, USA), and CSB (Santa Cruz Biotechnology, Santa Cruz, CA, USA). Western blot analysis using an anti‐GAPDH antibody (Santa Cruz Biotechnology) was also performed to validate equal loading among samples. Secondary HRP‐conjugated IgG antibodies were purchased from Sigma. Protein levels were visualized using an enhanced ECL substrate kit (Immobilon Crescendo Western HRP substrate, Merck, Millipore).

### 2.13 siRNA‐mediated CSB gene silencing

Inhibition of CSB expression in HDFs was performed as described in the past (Mavrogonatou et al., 2022) with the commercially available CSB siRNA (Santa Cruz Biotechnology), consisting of a pool of three target‐specific siRNAs designed to knock down CSB expression. Cells were transfected with 50 nM of either a predesigned scramble (5′‐UAAUGUAUUGGAACGCAUATT‐3′) or the CSB siRNA in serum‐free OptiMEM I medium using lipofectamine 2000 (Invitrogen). After a 5‐h incubation in a humidified atmosphere of 5% CO_2_ at 37°C, transfection medium was replaced by culture medium supplemented with 10% (v/v) FBS, and cells were further incubated for 48 h before any other treatment.

### 2.14 Co‐cultures

The ability of cancer cells for colony formation on top of HDFs was assessed as described in the past (Krtolica et al., 2001; Papadopoulou & Kletsas, 2011) with slight modifications. In brief, confluent cultures of young, resistant, and IS HDFs were overlaid with A431 cells (40 cells/well in a 6‐well plate) and further incubated for 12 days with a medium change every 4 days. At the end of the 12‐day incubation period, co‐cultures were washed once with PBS, fixed with 4% (v/v) formaldehyde for 10 min at room temperature, and stained with 1% (w/v) rhodanile blue in ddH_2_O for 30 min at room temperature. After removal of the remaining dye by washes with ddH_2_O, colonies were observed under an ECLIPSE Ts2 inverted microscope (Nikon) equipped with a Basler microscopy camera and software for image acquisition and analysis. Colonies' area in μm^2^ was calculated after outlining the perimeter of each colony using the *Closed Polygon* option of the *Measurement* tool.

### 2.15 In vivo tumorigenicity evaluation

To evaluate the role of HDFs in A431‐induced tumor growth in vivo, A431 cells were injected in three different spots in the back of 23 severe combined immunodeficiency (SCID) mice along with an equal number of young, resistant, or IS HDFs (100 μL/injection), as previously reported (Papadopoulou & Kletsas, 2011). Two weeks post‐injections, animals were sacrificed, and tumors were removed and weighed. All animal studies were conducted according to the institutional guidelines conforming to international standards under the approval of the ethical committee of the NCSR “Demokritos” Animal Facility (729592/27–07–2022).

### 2.16 Collection of conditioned medium and MMP activity assay

Conditioned medium was collected from subconfluent HDFs as described previously (Liakou et al., 2016), with slight modifications. Briefly, after three washes with phenol red‐free and serum‐free DMEM, HDFs grown in 100‐mm culture dishes were incubated with fresh phenol red‐free and serum‐free DMEM for 48 h. Conditioned medium was then collected and centrifuged (2000 ***g***/30 min) to be clarified from cell debris before its 10‐fold concentration with Vivaspin® 6 centrifugal concentrators with a molecular weight cut‐off of 3 kDa (Sartorius, Göttingen, Germany), aliquoted, and stored at −80°C until use.

MMP activity was determined using the fluorogenic substrate for MMP‐1, ‐2, ‐3, and ‐9 Dabcyl‐Gaba‐Pro‐Gln‐Gly‐Leu‐Glu‐(EDANS)‐Ala‐Lys‐NH2 (Cayman Chemical, Ann Arbor, MI, USA) at a final concentration of 10 μM (Kouroumalis et al., 2019; Liakou et al., 2016). After incubation at 37°C for 48 h in the dark, fluorescence was measured at 480 nm after excitation at 340 nm, using a Fluostar Optima microplate reader (BMG Labtech, Offenburg, Germany).

### 2.17 Statistical analysis

Data presented are the means ± standard deviation. Statistically significant differences were determined at the 95% confidence level (Student's *t* test for two‐sample and ANOVA, Tukey's test for multiple‐sample comparison). Analyses were performed and graphs/plots were created using GraphPad Prism version 5.0 (GraphPad Software, San Diego, CA, USA), Statgraphics Centurion software (Manugistics Inc., Dallas, TX, USA), the Bioconductor package *metaseqR* ^*2*^, and *matplotlib* for visualization with Python.

## 3 RESULTS

### 3.1 The effect of UVB irradiation on cell viability and senescence induction

**FIGURE 1:**
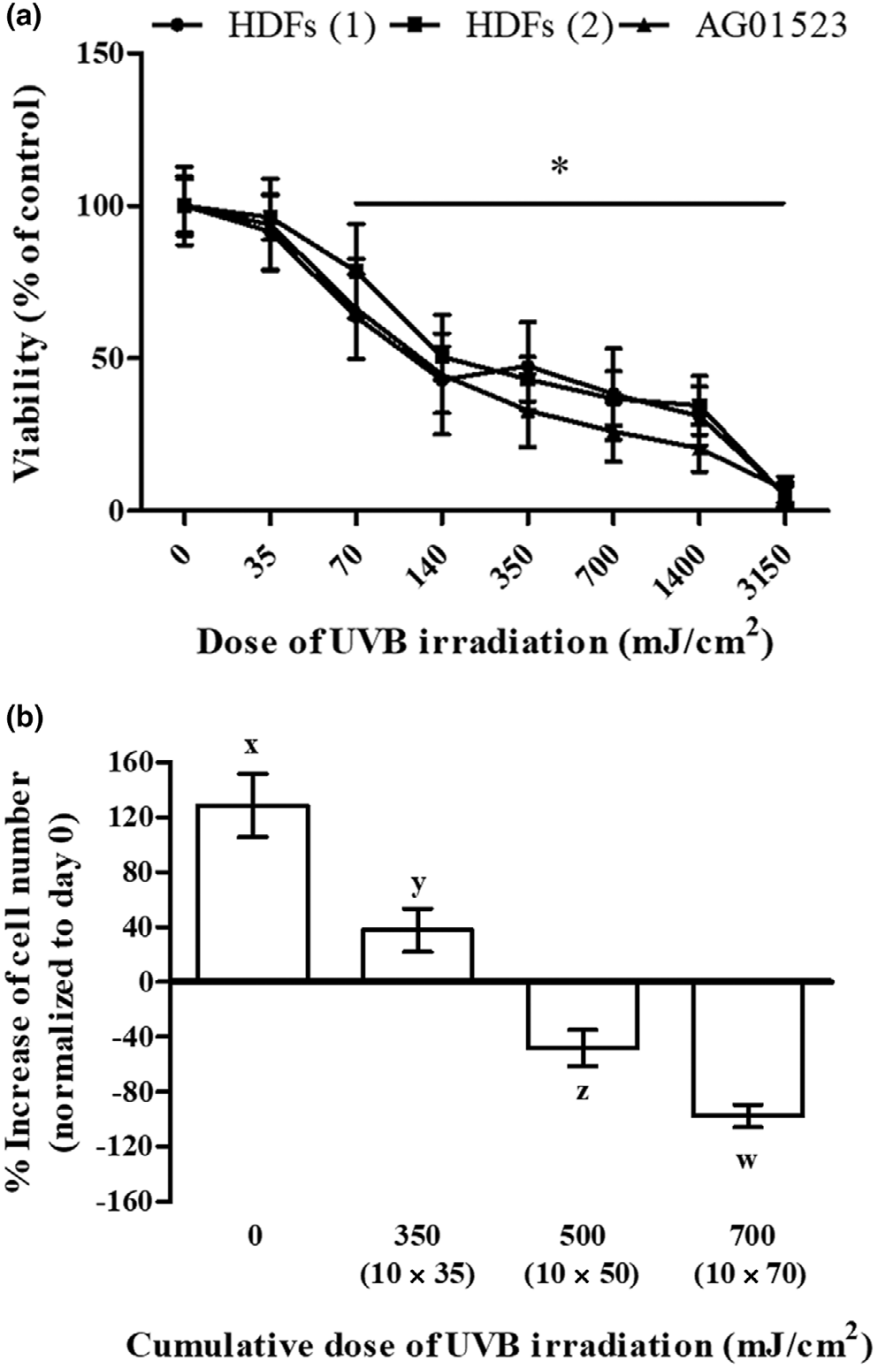
UVB irradiation is cytotoxic for human dermal fibroblasts (HDFs) in a dose‐dependent manner. (a) Primary HDFs from two different adult donors, as well as the commercially available cell line of foreskin fibroblasts AG01523, were exposed to UVB radiation doses ranging from 35 to 3150 mJ/cm^2^. After incubation at 37°C for 72 h, cells were detached by trypsinization, stained with neutral red, and measured using a hemocytometer. Unirradiated cells served as the control sample. Numerical values are the means ± standard deviations of two independent experiments conducted in duplicates. Asterisks denote statistically significant differences in comparison to unirradiated cells (Student's *t* test, *p* < 0.05). (b) Cells were exposed to various doses of UVB radiation (either 35, 50, or 70 mJ/cm^2^) twice a day for 5 consecutive days (cumulative UVB radiation dose of 350, 500, and 700 mJ/cm^2^, respectively). Unirradiated cells served as the untreated control. At the end of the ten doses' scheme (day 5), cells were detached by trypsinization, the number of viable cells was counted after staining with neutral red using a hemocytometer, and the obtained values were normalized to that of unirradiated cells at day 0. Statistically significant differences between any pair of means at the given UVB radiation dose are depicted by diverse letters (ANOVA, Tukey's test, *p* < 0.05).

We first studied the effect of UVB irradiation on human skin fibroblasts' viability. As can be seen in Figure 1a, UVB irradiation was cytotoxic for three normal fibroblast cell strains in a dose‐dependent manner, with an LD_50_ of 139.3 ± 16.5 mJ/cm^2^ and with 35 mJ/cm^2^ found to be the highest non‐cytotoxic irradiation dose tested. We next followed a classical protocol to establish premature senescence in HDFs proposed by others, that is, by exposing the cells to ten mild doses of UVB radiation (twice a day for 5 days) (Borlon et al., 2008; Debacq‐Chainiaux et al., 2005; Zhou et al., 2013). Prior to the application of this protocol for SIPS induction, a preliminary experiment assessing cell viability of HDFs at the end of the ten doses' scheme was conducted, using the maximal non‐cytotoxic dose of 35 mJ/cm^2^, as well as two higher (but still lower than the LD_50_) doses (50 and 70 mJ/cm^2^). As can be seen in Figure 1b, cells receiving the 10 × 35 mJ/cm^2^ irradiation scheme (cumulative dose of 350 mJ/cm^2^) displayed a significantly smaller increase in cell number than unirradiated cells, supporting a clear growth arrest. On the other hand, when the dose of 50 mJ/cm^2^ was used (cumulative dose of 500 mJ/cm^2^) a significant decrease of cell number was observed, showing a strong cytotoxic effect. This phenomenon was even more pronounced when the dose of 70 mJ/cm^2^ was used (cumulative dose of 700 mJ/cm^2^). Accordingly, the 10 × 35 mJ/cm^2^ irradiation scheme (unless otherwise stated) was used to provoke SIPS in HDFs in the rest of our experiments.

**FIGURE 2:**
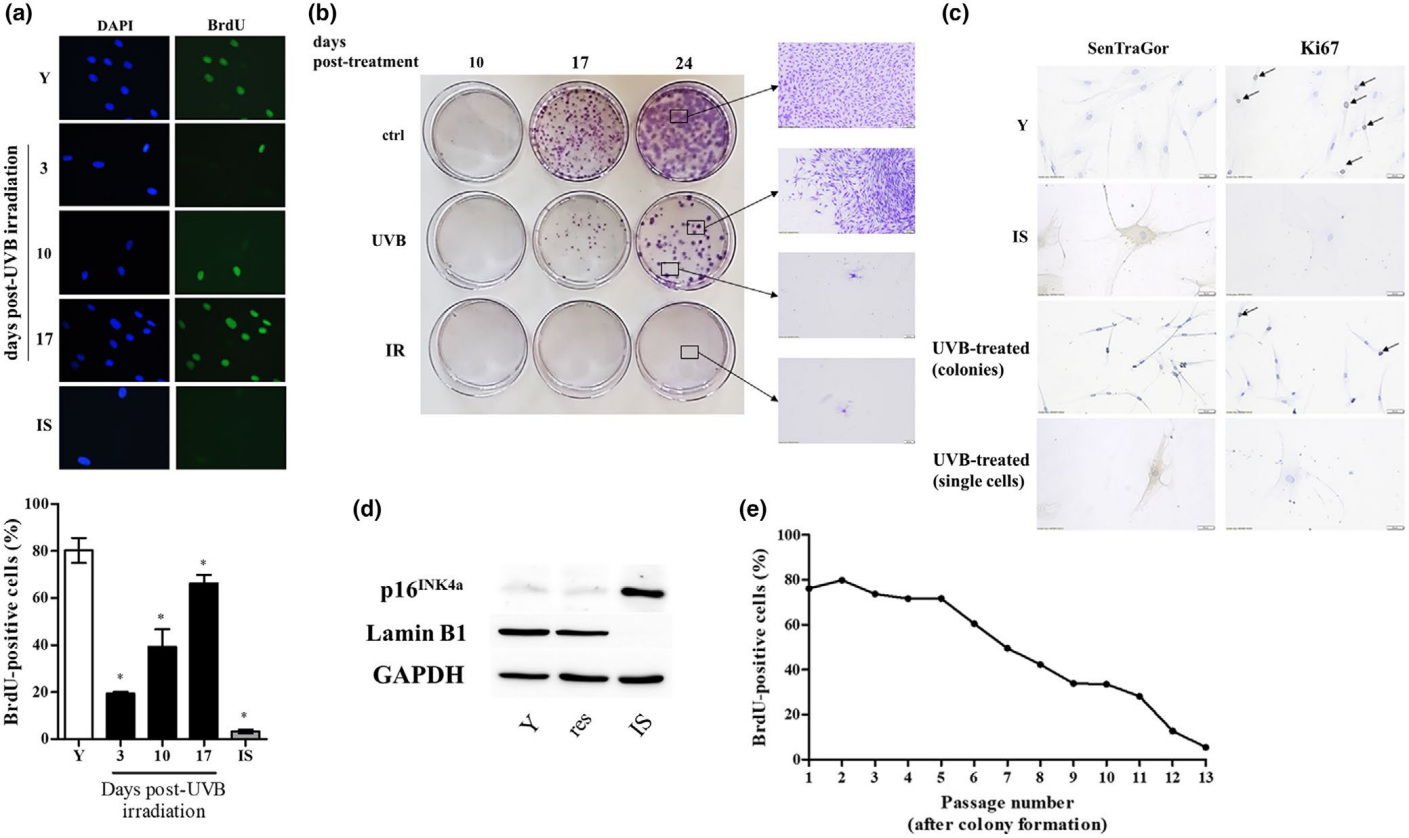
A subset of UVB‐treated human dermal fibroblasts (HDFs) regain their proliferative potential after an initial growth arrest with no signs of transformation. (a) HDFs were exposed to a 10 × 35 mJ/cm^2^ UVB irradiation scheme and they were allowed to attach onto glass coverslips for 3, 10, and 17 days post‐treatment before their labeling with 50 μM BrdU. After fixation, samples were stained with an anti‐BrdU‐FITC antibody, counterstained with DAPI, and observed under a fluorescence microscope. Unirradiated early‐passage (young, Y) cells served as the untreated control, while cells exposed to ionizing radiation served as the positive control for cellular senescence (ionizing radiation‐induced senescent cells, IS). BrdU incorporation is expressed as a % ratio of the number of BrdU‐positive nuclei to the number of DAPI‐positive nuclei. Microscopic pictures and means ± standard deviations in the graph are from a representative experiment out of three similar ones. Asterisks denote statistically significant differences in comparison to Y (Student's *t* test, *p* < 0.05). (b) Untreated HDFs (ctrl), HDFs exposed to a 10 × 35 mJ/cm^2^ UVB irradiation scheme (UVB) and HDFs exposed to ionizing irradiation (IR) were very sparsely plated and left to grow onto the surface of petri dishes for 10, 17, and 24 days. After fixation, colonies formed were stained with crystal violet. (c) Unirradiated early‐passage (Y), ionizing radiation‐induced senescent (IS) and exposed to a 10 × 35 mJ/cm^2^ UVB irradiation scheme (UVB‐treated) HDFs were allowed to attach onto glass coverslips for 10 days before their fixation and the staining of lipofuscin‐containing cells with the SenTraGor reagent and of proliferating cells with an anti‐Ki67 antibody. Representative microscopic pictures from one experiment out of three similar ones are depicted here. (d) Protein extracts from Y, resistant (res) and ionizing radiation‐induced senescent (IS) HDFs were subjected to western blot analysis with primary antibodies raised against p16^INK4a^ and lamin B1. Western blot analysis using an anti‐GAPDH antibody was also performed to validate equal loading among samples. Representative blots from three independent experiments are presented here. (e) Resistant UVB‐treated HDFs were serially subcultured, and nuclear BrdU incorporation was estimated at every passage post‐colony formation as described in (a). A representative experiment of three similar ones is shown here.

For the characterization of senescent cells, the proliferative potential of HDFs subjected to the 10 × 35 mJ/cm^2^ UVB irradiation scheme was monitored 3, 10, and 17 days post‐irradiation by estimating nuclear BrdU incorporation. As shown in Figure 2a, 3 days post‐UVB irradiation, HDFs were significantly arrested in comparison to young untreated cells, similarly to previously published results, retaining though a higher ability for BrdU incorporation than that of ionizing radiation‐induced senescent (IS) cells. Besides, these arrested cultures, as a whole, expressed several established biomarkers of senescence (Bulbiankova et al., 2023; Mavrogonatou et al., 2021, 2023), such as p16^INK4a^, p21^WAF1^, ICAM‐1, IL‐6, IL‐8, MMP‐1, and MMP‐13 up‐regulation, as well as collagen I α down‐regulation, while the majority of the cells were positively stained with SenTraGor for lipofuscin accumulation (Evangelou et al., 2017) (Figure S1). However, a follow‐up observation at days 10 and 17 post‐UVB irradiation indicated a progressive increase of the percentage of BrdU incorporation and lamin B1 expression, along with the decrease of p16^INK4a^ protein levels, all markers highly approaching at day 17 post‐UVB irradiation the levels of untreated cells (Figure 2a and Figure S2). This finding indicates that UVB‐irradiated cultures were not composed (at least not solely) by senescent cells and contained a fraction of proliferating cells. This is in contrast to HDFs exposed to ionizing radiation that entirely and permanently lost their proliferative potential and were driven to senescence (IS cells in Figure 2a), as previously shown for other normal fibroblasts (Liakou et al., 2016; Papadopoulou & Kletsas, 2011).

In order to further characterize UVB‐treated HDFs, we performed a colony assay (Figure 2b). Untreated fibroblasts were able to form visible colonies even from day 10, the number and size of which increased to a greater extent at day 17 and 24. In contrast, cell populations exposed to UVB radiation (10 × 35 mJ/cm^2^) were mainly characterized by single cells and sporadically proliferating cells at day 10 post‐UVB irradiation; at days 17 and 24 post‐UVB irradiation, cultures became mixed, that is, they composed of a low number of colonies (lower than that of untreated cells) and single, non‐proliferating cells. Similar results were also observed in two additional normal skin fibroblast cell strains, as well as in normal lung fibroblasts, indicating that this is a general phenomenon for fibroblasts (Figure S3). On the other hand, IS cells formed no colonies, validating their established senescent phenotype (Figure 2b).

We then proceeded with the characterization of repeatedly UVB‐treated HDFs originating from the two distinct groups (i.e., single cells and cells forming colonies) in terms of senescence markers, in comparison to their untreated and ionizing radiation‐treated counterparts. As shown in Figure 2c, unirradiated (young) cells displayed positive staining for the proliferation marker Ki67 and were negative for the marker of lipofuscin accumulation in senescent cells when stained with SenTraGor (Evangelou et al., 2017). The reverse staining pattern was, as expected, observed in IS cells that were Ki67‐negative and SenTraGor‐positive. On the other hand, 10, 17, and 24 days post‐UVB irradiation, single cells were found to be Ki67‐negative and SenTraGor‐positive, while cells that gave rise to colonies had the profile of unirradiated cells (Figure 2c). Thus, it seems that repeated exposures of HDFs to UVB radiation (i.e., following the 10 × 35 mJ/cm^2^ irradiation scheme) result in a mixed population consisting of prematurely senescent cells, which cohabit with cells that, after an initial growth arrest, resist premature senescence and resume a proliferative state. This hypothesis was confirmed by the observation that after colony formation, this latter cell population (hereafter referred to as “resistant” cells), in contrast to IS cells, was negative for two classical markers of senescent fibroblasts, showing no p16^INK4a^ up‐regulation and no lamin B1 down‐regulation (Figure 2d).

To test the possibility that resistant UVB‐treated HDFs were rendered immortalized or acquired any cancer cells' traits, we performed serial subculturing of the cells. As shown in Figure 2e, resistant UVB‐treated HDFs were not driven to immortalization, but they instead progressively lost their ability to proliferate, ultimately reaching replicative senescence after a certain number of passages, which is indicative of a normal cell population. In addition, in contrast to human keratinocytes that have been reported to become tumorigenic after their exposure to repetitive UVB irradiation (Tyagi et al., 2015), resistant HDFs were not found to be tumorigenic, since they did not form any tumors when injected subcutaneously in the back of SCID mice, even 4 months post‐injection (data not shown).

### 3.2 Molecular characterization of resistant UVB‐treated HDFs

**FIGURE 3:**
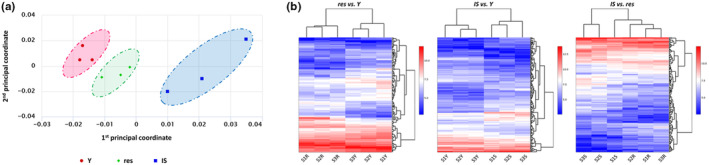
Resistant HDFs constitute a separate population from both young and ionizing radiation‐induced senescent cells based on their transcriptional profile. (a) Total RNA was extracted from young (Y), resistant (res), and ionizing radiation‐induced senescent (IS) HDFs and was then used for RNA‐seq analysis. Levels of similarities/dissimilarities and clustering of samples are visualized by a multidimensional scaling (MDS) plot of RNA‐sequencing datasets corresponding to Y, res, and IS HDFs. (b) Differentially expressed genes (DEGs) heatmaps depicting clustering of samples according to their expression values after normalization and statistical testing and possible clusters of co‐expressed genes.

Next, we performed an RNA‐seq analysis to shed light on the gene expression profile of resistant UVB‐treated HDFs in comparison to young and IS cells. The multidimensional scaling (MDS) plot of the nine sequencing datasets (three sequencing datasets per condition corresponding to young, resistant, and IS HDFs) revealed a clear separation of young, resistant, and IS cells in three groups (Figure 3a), while differentially expressed genes (DEGs) heatmaps showed the formation of two distinct groups when comparing all contrasts by pairs (Figure 3b). The combined analysis performed by *metaseqR* ^*2*^ resulted in 1156 statistically significant DEGs (of which 657 up‐regulated and 499 down‐regulated) in IS versus young cells; in 569 statistically significant DEGs (of which 301 up‐regulated and 268 down‐regulated) in resistant versus young cells; and in 812 statistically significant DEGs (of which 508 up‐regulated and 304 down‐regulated) in IS versus resistant cells (Tables S1–S3).

### 3.3 Functional characterization of resistant UVB‐treated HDFs

**FIGURE 4:**
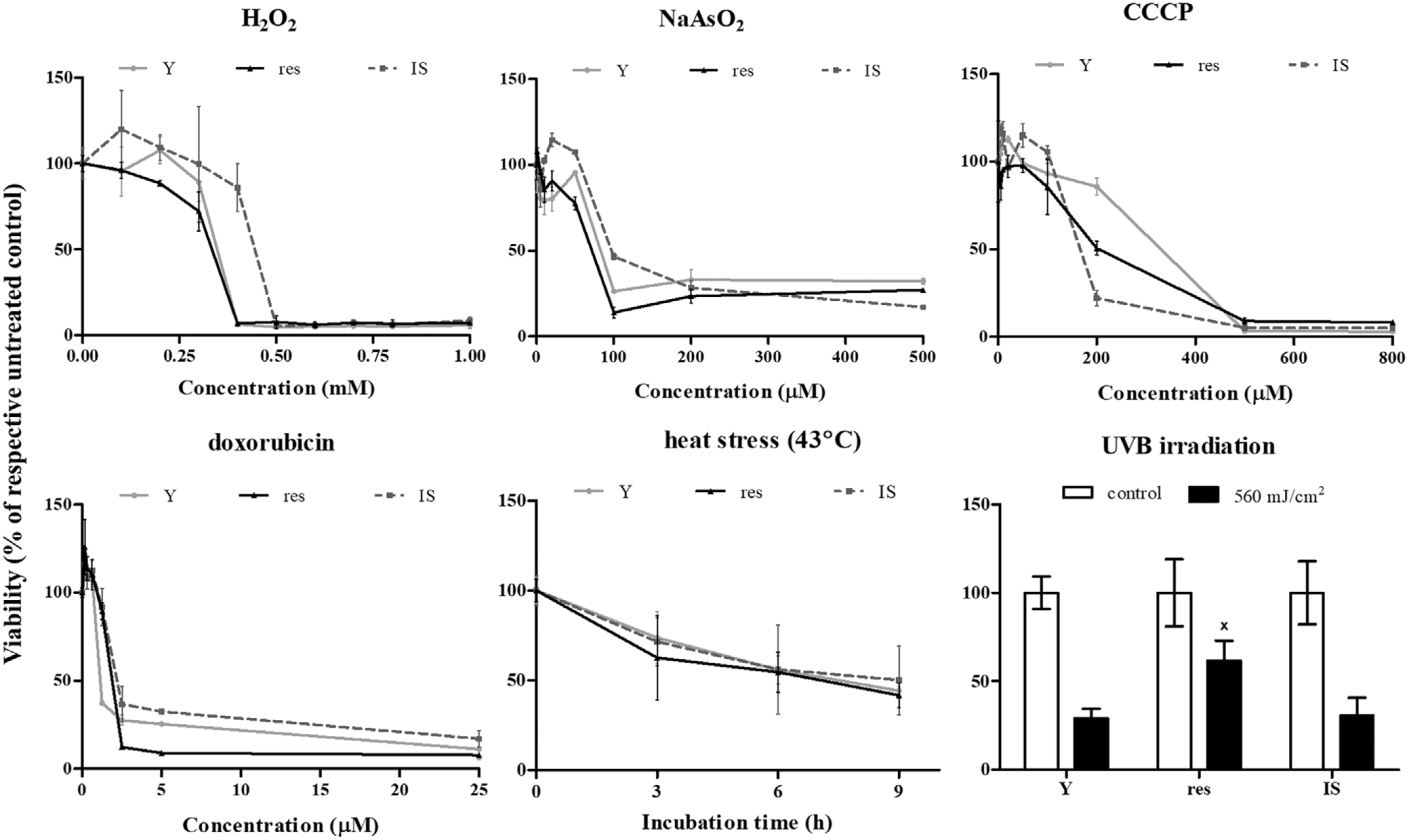
Tolerance of young (Y), resistant (res), and ionizing radiation‐induced senescent (IS) HDFs toward various types of stresses. Cells were treated with 0–1000 μΜ H_2_O_2_, 0–500 μΜ NaAsO_2_, 0–800 μΜ CCCP, 0–25 μΜ doxorubicin and exposed to 43°C for 3, 6, and 9 h or to a single dose of UVB radiation (560 mJ/cm^2^). Cell viability after staining of the cells with the vital dye neutral red was estimated 72 h post‐treatment. Untreated cells served as the control sample. Numerical values are the means ± standard deviations of at least two independent experiments. Not sharing a common letter denotes statistically significant differences between any pair of means at a particular concentration, time‐point, or dose (ANOVA, Tukey's test, *p* < 0.05).

Given that resistant HDFs had already coped an intense stress (that of repeated exposures to UVB radiation), we tested the possibility of a general resilience of these cells toward a number of known stresses. To this end, young, resistant, and IS cells were exposed to three different forms of oxidative stress (H_2_O_2_, NaAsO_2_, and the mitochondrial uncoupler CCCP), to the direct‐acting genotoxic agent doxorubicin or to heat stress. As can be seen in Figure 4, these stresses were cytotoxic for all young, resistant, and IS HDFs in a dose‐ or time‐dependent manner, and no major difference in their response was observed among the three cell populations tested. On the contrary, resistant HDFs were found to tolerate significantly better their treatment with a high dose of UVB radiation (560 mJ/cm^2^) than young and IS cells.

**FIGURE 5:**
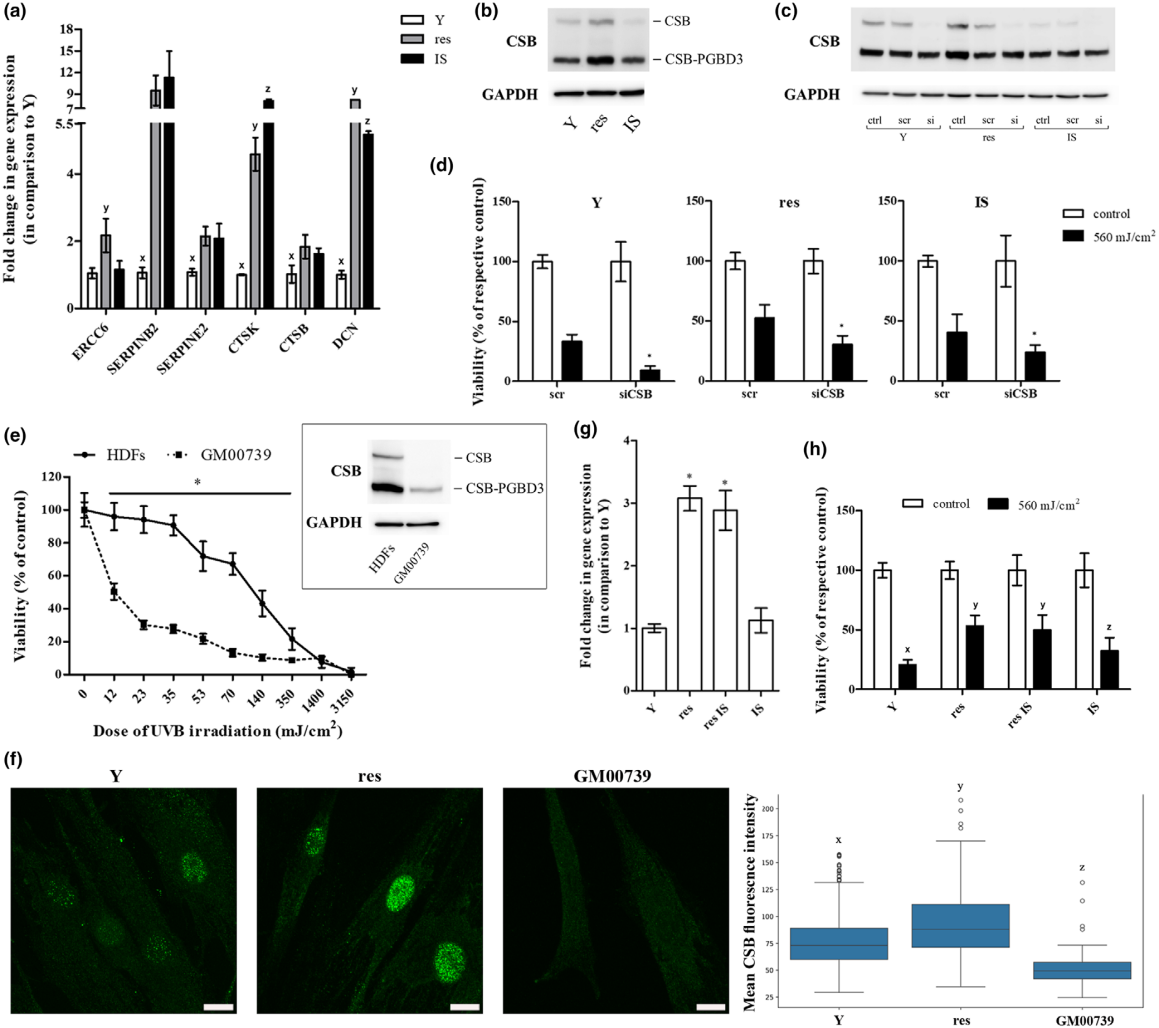
Cockayne syndrome group B (CSB) plays a crucial role in the protection of HDFs toward UVB‐mediated cytotoxicity. (a) Total RNA was extracted from young (Y), resistant (res), and ionizing radiation‐induced senescent (IS) HDFs and was then used for RT‐qPCR analysis. Glyceraldehyde‐3‐phosphate dehydrogenase (GAPDH) served as the reference gene. Numerical values are the means ± standard deviations of at least two independent experiments conducted in duplicates. Samples not sharing a common letter per gene tested are significantly different (ANOVA, Tukey's test, *p* < 0.05). (b) Total protein extracts from young (Y), resistant (res), and ionizing radiation‐induced senescent (IS) HDFs were subjected to western blot analysis with a primary antibody raised against the N‐terminus of CSB protein, able to hybridize with both the functional CSB and the CSB‐piggyBac fusion protein (CSB‐PGBD3). Western blot analysis for GAPDH served as the loading control. A representative blot from three independent experiments is shown here. (c) Young (Y), resistant (res), and ionizing radiation‐induced senescent (IS) HDFs were transfected with 50 nM of either a predesigned scramble (scr) or the CSB siRNA (si) before total protein extraction 48 h post‐transfection and western blot analysis for CSB protein. GAPDH served as the loading control. A representative blot from at least three independent experiments is depicted. (d) Transfected with CSB siRNA (siCSB) young (Y), resistant (res), and ionizing radiation‐induced senescent (IS) HDFs were exposed to a high dose of UVB radiation (560 mJ/cm^2^), stained with neutral red 72 h post‐treatment and measured. Cells transfected with scramble (scr) served as the control sample. Values in the graph are the means ± standard deviations of at least two independent experiments conducted in quadruplicates. Asterisks denote statistically significant differences in comparison to the respective scramble (Student's *t* test, *p* < 0.05). (e) Primary HDFs and dermal fibroblasts from a Cockayne syndrome patient carrying a mutation in the ERCC6 gene (GM00739) were exposed to UVB radiation doses ranging from 12 to 3150 mJ/cm^2^. After incubation at 37°C for 72 h, cells were detached, stained with neutral red, and counted. Unirradiated cells served as the control sample. Numerical values are the means ± standard deviations of two independent experiments conducted in duplicates. Asterisk denotes statistically significant differences in comparison to normal HDFs at the given UVB radiation doses (Student's *t* test, *p* < 0.05). In the insert, the expression profile of CSB and CSB‐PGBD3 of HDFs and GM00739 dermal fibroblasts is presented. (f) Young (Y) untreated and resistant (res) HDFs were allowed to attach onto glass coverslips, fixed, stained with an anti‐CSB antibody, counterstained with DAPI, and observed under a confocal laser scanning microscope. Representative images of the CSB channel maximum intensity projection (MIP) from three independent experiments are shown here (Scale bar: 15 μm). GM00739 cells not expressing a functional CSB protein served as the negative control. The mean CSB fluorescence intensity of Y, res and GM00739 HDFs is presented in the box plot. Statistically significant differences between any pair of samples are marked by dissimilar letters (ANOVA, Tukey's test, *p* < 0.05). (g) RNA was extracted from young (Y), resistant (res), resistant‐rendered senescent after exposure to ionizing radiation (res IS), and ionizing radiation‐induced senescent (IS) HDFs before RT‐qPCR analysis for ERCC6 gene expression. GAPDH served as the reference gene. Values in the graph are the means ± standard deviations of two experiments conducted in duplicates. Samples sharing a common symbol are not significantly different (ANOVA, Tukey's test, *p* < 0.05). (h) Young (Y), resistant (res), resistant rendered senescent after exposure to ionizing radiation (res IS) and ionizing radiation‐induced senescent (IS) HDFs were exposed to 560 mJ/cm^2^ of UVB radiation and measured 72 h post‐irradiation after their staining with neutral red. Unirradiated cells served as the control sample. Values in the graph are the means ± standard deviations of four independent experiments in quadruplicates. Dissimilar letters denote statistically significant differences between any pair of means (ANOVA, Tukey's test, *p* < 0.05).

In an attempt to shed light on the molecular characteristics of resistant HDFs responsible for their endurance toward UVB stress, relative mRNA levels of selected genes that were found to be up‐regulated in resistant versus young cells after the functional annotation and classification of RNA‐seq data using the GO term *response to stress* were assessed in young, resistant, and IS HDFs by RT‐qPCR analysis (Figure 5a). From these genes, only ERCC6—encoding DNA excision repair protein ERCC6, also called CSB protein—was shown to be exclusively significantly up‐regulated in resistant cells, as compared to young and IS cells. CSB up‐regulation in resistant cells was also verified at the protein level (Figure 5b). To further investigate the implication of CSB in the response of HDFs toward UVB irradiation, we used an siRNA approach to efficiently knock down CSB protein expression in HDFs (Figure 5c). siRNA‐mediated CSB loss‐of‐expression rendered all young, resistant, and IS HDFs significantly more susceptible to a high dose (560 mJ/cm^2^) of UVB radiation (Figure 5d), indicating that indeed CSB plays a crucial role in the protection of HDFs toward UVB‐mediated cytotoxicity. In the same vein, dermal fibroblasts from a Cockayne syndrome patient (carrying a mutation in the ERCC6 gene resulting in the loss of a functional CSB protein) were dramatically more vulnerable to UVB irradiation, in addition to the formation of a lower number of colonies after their repetitive exposure to their highest non‐cytotoxic UVB radiation dose, compared to HDFs from normal donors (Figure 5e and Figure S4).

Interestingly, a single UVB radiation dose of 35 mJ/cm^2^ was not found to enhance ERCC6 gene expression (Figure S5A). Furthermore, ERCC6 expression levels in the whole irradiated HDF population were similar to those of unirradiated cells right after the end of the repeated UVB radiation doses' scheme (10 × 35 mJ/cm^2^) and only several days post‐irradiation—when resistant cells dominated in the culture—a significant ERCC6 up‐regulation, as shown by CSB overexpression, was observed (Figure S5B,C). These findings suggest that resistant HDFs most probably represent an already existing subpopulation overexpressing this protein. Indeed, observation of CSB‐immunostained nuclei under a confocal microscope, followed by quantification of the distribution of CSB fluorescence intensity per nucleus normalized by the nucleus area revealed the occurrence of three distinct clusters of cells in terms of mean CSB fluorescence intensity (low, medium, and high) in the initial population of untreated HDFs (Figure 5f and Figure S6). Moreover, in accordance with the higher CSB expression levels observed in resistant cells as a whole, the high mean CSB fluorescence intensity cluster was found to be amplified in the population of resistant HDFs (Figure S6). In favor of ERCC6 overexpression being an inherent characteristic of this particular subset of HDFs, resistant HDFs that were subsequently exposed to ionizing radiation were shown to undergo senescence (from this point forward referred to as “resistant IS” cells), displaying ERCC6 expression levels similar with those of untreated resistant cells and higher than those of young and IS cells (Figure 5g). Of note—and in agreement to the implication of CSB in the observed resilience toward UVB stress shown in Figure 5d—“resistant IS” cells were found to be more tolerant toward UVB irradiation than young and IS cells and equally protected from UVB‐mediated cytotoxicity with resistant HDFs (Figure 5h).

### 3.4 Role of resistant UVB‐treated HDFs in tumor promotion

**FIGURE 6:**
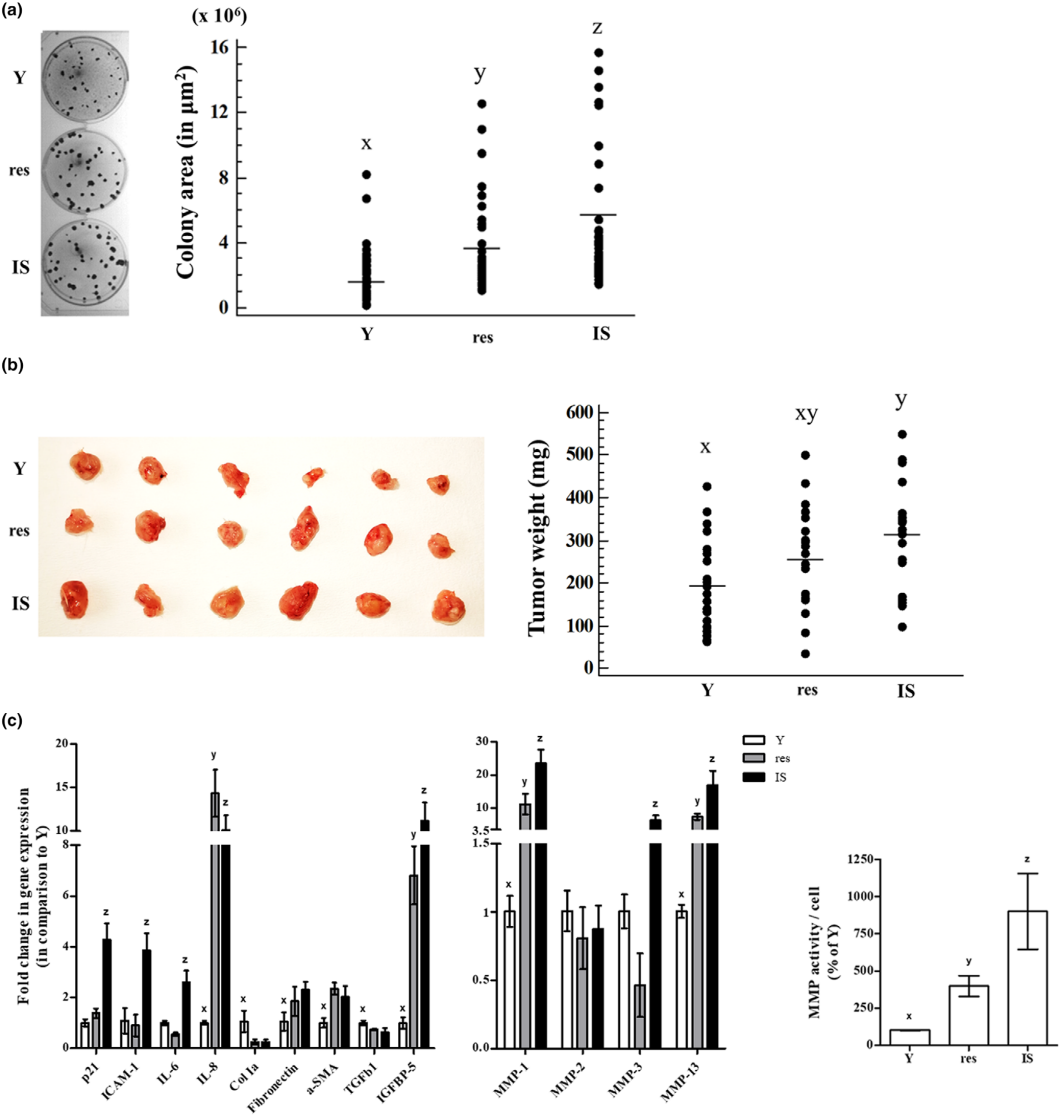
Resistant HDFs promote cancer cells' growth in vitro and in vivo. (a) Confluent cultures of young (Y), resistant (res), and ionizing radiation‐induced senescent (IS) HDFs were overlaid with A431 cells and further incubated for 12 days. At the end of the 12‐day incubation period, co‐cultures were stained with rhodanile blue, observed under the microscope, and colonies' area in μm^2^ was calculated. Dissimilar letters denote statistically significant differences among compared samples (ANOVA, Tukey's test, *p* < 0.05). (b) A431 cells were injected in three different spots in the back of severe combined immunodeficiency (SCID) mice along with an equal number of young (Y), resistant (res), or ionizing radiation‐induced senescent (IS) HDFs. Animals were sacrificed 2 weeks post‐injections, tumors were removed, and weighed. Photographs of tumors excised from 6 representative animals are presented here. Groups not sharing a common letter are considered significantly different (ANOVA, Tukey's test, *p* < 0.05). (c) Total RNA was extracted from young (Y), resistant (res), and ionizing radiation‐induced senescent (IS) HDFs and was then used for RT‐qPCR analysis using specific primers for the designated genes. Glyceraldehyde‐3‐phosphate dehydrogenase (GAPDH) was used as the reference gene. Numerical values are the means ± standard deviations of at least two independent experiments conducted in duplicates. Dissimilar letters denote statistically significant differences between any pair of means for every gene tested (ANOVA, Tukey's test, *p* < 0.05). Conditioned media of young (Y), resistant (res) and ionizing radiation‐induced senescent (IS) HDFs were collected and concentrated 10‐fold, before the determination of MMP activity using the fluorogenic substrate Dabcyl‐Gaba‐Pro‐Gln‐Gly‐Leu‐Glu‐(EDANS)‐Ala‐Lys‐NH2. After incubation at 37°C for 48 h in the dark, fluorescence was measured at 480 nm after excitation at 340 nm. Enzymatic activity per cell was expressed as a % ratio of that of Y cells. Numerical values are the means ± standard deviations of three independent experiments conducted in duplicates. Dissimilar letters denote statistically significant differences in the enzymatic activity among the three compared samples (ANOVA, Tukey's test, *p* < 0.05).

It is well recognized that UVB irradiation is a risk factor for skin carcinogenesis and, in addition to that, senescent cells can support the growth of cancer cells in vitro and in vivo (Krtolica et al., 2001; Papadopoulou & Kletsas, 2011). Accordingly, to further characterize resistant HDFs, we first explored their potential to subserve the growth of the human squamous cell carcinoma A431 cells in vitro. As shown in Figure 6a, sparsely seeded A431 cells onto confluent cultures of resistant HDFs formed colonies significantly larger than those formed onto cultures of young HDFs but significantly smaller than those formed onto IS HDFs' cultures, confirming once again that resistant UVB‐treated cells constitute a distinct population from young and IS HDFs.

Furthermore, when A431 cells were subcutaneously injected in the back of SCID mice along with young, resistant, or IS HDFs, IS cells promoted a significant increase in tumor growth in comparison to young cells, as expected (Krtolica et al., 2001, Papadopoulou & Kletsas, 2011). On the other hand, the mean weight of tumors formed by A431 cells when co‐injected with resistant HDFs was in‐between the tumors formed in the presence of young and IS cells (Figure 6b).

It has been well established that senescent cells possess a senescence‐associated molecular profile coding for their arrested, pro‐inflammatory, and catabolic phenotype. Moreover, among the typical senescence‐related markers (including cell cycle regulators, inflammatory cytokines, as well as regulators of ECM synthesis, and degradation), several categories of SASP factors (e.g., ILs, growth factors, growth factor carriers, and MMPs) have been reported to be connected with tumor promotion (Niklander et al., 2021; Schmitt et al., 2022; Ye et al., 2023). Thus, to further characterize resistant HDFs, we assessed the transcriptional profile of selected genes encoding senescence‐related markers in young, resistant, and IS HDFs (Figure 6c). Notably, depending on the gene of interest, resistant cells occasionally expressed a transcriptional profile resembling either that of young (e.g., for p21^WAF1^, ICAM‐1, IL‐6, MMP‐3) or IS (e.g., for IL‐8, Col1a, fibronectin, α‐SMA, TGFβ1, IGFBP‐5, MMP‐1, MMP‐13) cells. By contrast to SASP factors that may directly promote cancer growth due to a mitogenic effect (i.e., growth factors), increased secretion of MMPs by senescent cells has been previously shown to indirectly facilitate cancer expansion by creating a permissive microenvironment due to the degradation of ECM (Liu & Hornsby, 2007; Papadopoulou & Kletsas, 2011). For that reason, we also assessed the activity of MMPs secreted by young, resistant, and IS HDFs using a FRET peptide‐based MMP assay with a substrate for MMP‐1, ‐2, ‐3, and ‐9. As can be seen in Figure 6c, the medium conditioned by resistant HDFs had increased MMP activity compared to that of young cells and decreased in comparison to IS cells. All the above verify that resistant HDFs constitute a separate population from both young and IS cells, with an intermediate phenotype which is reflected in their effect on skin homeostasis.

## 4 DISCUSSION

The skin is the largest organ of the human body, which is continuously exposed to a variety of environmental stressors. Among these, solar UV irradiation may have detrimental effects on skin tissue homeostasis, including photodamage, photoaging, and photocarcinogenesis (Ananthaswamy & Pierceall, 1990; Fisher et al., 1996; Gilchrest, 2013; Ley, 1985; Scharffetter‐Kochanek et al., 2000). From the two UV fractions reaching the surface of the earth, UVB radiation is much more energetic than UVA and, although largely absorbed by the epidermis, the quantity attaining dermal papilla is sufficient to negatively affect embedded fibroblasts. On one hand, selective loss of fibroblasts from the upper dermis in human and mouse skin has been documented as a response to acute and chronic exposure to UVB radiation (Mavrogonatou et al., 2022; Rognoni et al., 2021). On the other hand, repeated mild treatment with UVB radiation has been reported by several groups to result in HDFs' premature senescence (Cavinato et al., 2017; Chainiaux et al., 2002; Greussing et al., 2013).

In order to study UVB‐mediated SIPS in HDFs, we assessed the maximal *non‐cytotoxic* UVB radiation dose, which was estimated to be approx. 35 mJ/cm^2^. This dose was in agreement with our previous work (Mavrogonatou et al., 2022) and very close to those reported in other studies on UVB‐mediated cytotoxicity (Burón et al., 2022; Hao et al., 2019; Ryoo et al., 2001; Zeng et al., 2017). We then repeatedly exposed HDFs to 35 mJ/cm^2^ of UVB radiation following an established protocol for SIPS induction (i.e., twice a day for 5 days). In accordance to previous studies, 3 days post‐last exposure to UVB irradiation, we observed that HDFs had a significantly reduced proliferative potential and displayed a senescent‐like molecular profile (Borlon et al., 2007, 2008; Debacq‐Chainiaux et al., 2005, 2006; Zhou et al., 2013). Notably, each separate dose, as well as the cumulative UVB radiation dose received by the cells in our study, was much lower than those used in most previous studies following a similar UVB‐induced SIPS protocol, in which applied UVB radiation doses referred to as *subcytotoxic* were up to one order of magnitude higher (Borlon et al., 2008; Greussing et al., 2013; Lago & Puzzi, 2019; Zhou et al., 2013). However, it is worth mentioning that UVB‐mediated SIPS induction has been also reported to occur in human skin fibroblasts exposed to a lower than ours total UVB radiation dose (50 mJ/cm^2^) 3 and 6 days after treatment of the cells (Chen et al., 2008; Permatasari et al., 2014).

Surprisingly, a follow‐up observation of UVB‐treated cultures in our study revealed a progressive increase of the percentage of proliferating cells, as well as a progressive loss of p16^INK4a^ up‐regulation and lamin B1 down‐regulation at longer time points, reaching almost the levels of untreated HDFs 17 days post‐UVB irradiation. These cultures were found to be mixed, containing at the same time both senescent (incapable of proliferating, thus remaining single) and senescence‐resistant (resuming proliferation and forming colonies) cells. We suggest the designation “resistant” to describe these cells, since they do not fall within any of the already proposed terms in the literature. In contrast to “escape” cells that reacquire replication potency after a period of prolonged senescence (Evangelou et al., 2023; Reimann et al., 2024; Zampetidis et al., 2021) and diverging from “bypass” cells that evade senescence and undergo malignant transformation (Evangelou et al., 2023; Reimann et al., 2024) or enter an extended lifespan terminated by a crisis or crisis‐like state (Brown et al., 1997; Wei et al., 2003), resistant HDFs were shown to circumvent UVB‐mediated SIPS and to remain normal, as they underwent replicative senescence after several passages and were unable to form any tumors when injected subcutaneously in the back of SCID mice. To the best of our knowledge, the emergence of a subpopulation of HDFs resisting UVB‐mediated SIPS has not been described before. In the same direction, though, the senescence‐like traits observed in HDFs exposed to PUVA (8‐methoxypsoralen followed by UVA irradiation) (Herrmann et al., 1998) were subsequently proved to be fully reversible 100 to 130 days post‐PUVA treatment, suggesting that these reminiscent of cellular senescence morphological and functional alterations rather represented a long‐term transient phenocopy of senescence than an established senescent phenotype (Ma et al., 2002). It is also worth mentioning that, in accordance to our observations in UVB‐resistant HDFs, neither immortalization nor transformation have been accounted for the recovered growth capacity of PUVA‐treated HDFs, as well (Ma et al., 2002). Furthermore, it is interesting to note that a considerable amount of time has been shown to be needed for the manifestation of a full senescent phenotype, even as a response to excessive genotoxic stimuli: senescence has not been observed in human fibroblasts before 10–14 days after their exposure to ~50 Gy of ionizing radiation (Naka et al., 2004; Papadopoulou & Kletsas, 2011), while expression of the well‐defined biomarker of senescence/aging p16^INK4a^ has been shown to increase in normal human fibroblasts 30 days after their treatment with the DNA‐damaging agent bleomycin (Robles & Adami, 1998) and in the tissues of γ‐irradiated mice not before 4 weeks post‐irradiation (Le et al., 2010). Taken together with these data, our findings reinforce the notion that a long‐term observation of treated cultures is required to avoid misconceiving transient stress‐induced responses with SIPS and thus safely and accurately declare a cell population as senescent.

Further characterization of resistant HDFs using RNA‐seq analysis revealed that they constitute a distinct population. Given that this distinct population emerged after repeated non‐cytotoxic UVB treatment, we wondered whether tolerance to UVB irradiation was obtained in the framework of an adaptive response ascribing to the cells a universal resistance against various stressors (of direct or indirect genotoxic nature). In this vein, cells pre‐treated with UV irradiation, a chemical carcinogen, or hyperosmotic stress have been reported to more rapidly identify and adequately repair newly introduced DNA damage (Liu & Rainbow, 2004; Mavrogonatou & Kletsas, 2009; Protić et al., 1988). However, resistant cells described here showed no higher robustness toward any other stressor tested and were found to be more resilient than young or IS cells only against an extra treatment with an intense UVB radiation dose. This could be attributed either to the gradual development of a UVB‐specific resistance mechanism or to an inherent molecular trait carried by a pre‐existing subpopulation in the original cell culture, from which resistant HDFs may have expanded.

To examine this hypothesis and further elucidate the mechanism underlying UVB resistance in HDFs, we directed our attention to selected genes that were found to be up‐regulated in resistant versus young cells after the classification of the functionally annotated RNA‐seq data using the GO term *response to stress*. Validation with RT‐qPCR analysis verified SERPINB2, SERPINE2, CTSK, CTSB, and DCN up‐regulation in both UVB‐resistant and IS HDFs. In accordance, SERPINB2, CTSK, and DCN have been previously reported to be up‐regulated in UVB‐induced senescent HDFs (Greussing et al., 2013). Most importantly, our analysis revealed one gene (ERCC6) that was solely up‐regulated in resistant compared to young cells and not in IS cells, at the mRNA and protein levels. ERCC6 encodes CSB protein, a SWI/SNF‐like DNA‐dependent ATPase that can wrap DNA and remodel chromatin, being required for transcription‐coupled DNA repair (TCR) (Beerens et al., 2005; Citterio et al., 2000; Newman et al., 2006), but also functioning as a master switch factor that can selectively influence the transcription of specific sets of genes by allowing RNA polymerase II to initiate their RNA synthesis (Frontini & Proietti‐De‐Santis, 2009; Selby & Sancar, 1997). Notably, p53‐responsive genes have been shown not to require CSB for their transcription (Proietti‐De‐Santis et al., 2006). On the other hand, CSB—by being part of an E3 ubiquitin ligase complex—has been reported to control p53 levels through protein ubiquitination and degradation in an Mdm2‐dependent manner (Latini et al., 2011; Paccosi & Proietti‐De‐Santis, 2021; Spyropoulou et al., 2021), while HIF‐1‐mediated up‐regulation of CSB under hypoxic stress has been reported to redirect cells toward survival and proliferation instead of cell cycle arrest and cell death by competing the binding of the co‐activator p300 to p53 and by therefore decreasing p53 transactivation activity (Filippi et al., 2008). We can thus speculate that observed UVB tolerance of ERCC6‐overexpressing HDFs in our study is most probably attributed to a CSB‐induced mechanism, not involving p53 activation, which could possibly explain why resistant cells did not show any resilience toward other genotoxic insults beyond UVB irradiation.

The role of CSB in conferring cytoprotection to HDFs from UVB irradiation was confirmed by siRNA‐mediated knocking down of its expression. We showed that CSB loss‐of‐expression led to a decreased resistance of all young, resistant, and IS HDFs, which were characterized by a significant reduction in their viability after exposure to UVB irradiation, in accordance with the UV hypersensitivity and defective TCR of UV damage observed in cells from UV‐sensitive syndrome patients, owed to the absence of CSB (Horibata et al., 2004). Interestingly, even though resistant cells showed a concomitant overexpression of the CSB and the CSB‐PGBD3 fusion protein (an alternatively spliced form of the first five exons of the gene with no ATPase motifs, due to the integration of a piggyBac3 transposable element into intron 5), here we showed that siRNA‐mediated knocking down of the functional CSB protein alone (leaving the expression levels of CSB‐PGBD3 fusion protein unaffected) was sufficient to render cells more vulnerable toward UVB irradiation, in contrast to the reported implication of CSB‐PGBD3 fusion protein in DNA repair in CSB‐null cells (Bailey et al., 2012). Nevertheless, our finding is in agreement with a previous report in which it was shown that—among various CSB domains tested—critical for preventing UV‐induced apoptosis is the integrity of the ATPase domain (Balajee et al., 2000). In favor of the key role of CSB in UVB cytoprotection, as well as in the resistance to UVB‐induced senescence, dermal fibroblasts from a Cockayne syndrome patient with a mutation in the ERCC6 gene resulting in the loss of a functional CSB protein, but normally expressing the fusion protein were shown here to be dramatically more vulnerable to an acute high UVB irradiation dose and to form a much lower number of colonies after repetitive UVB exposure, compared to HDFs from normal donors. The latter is in accordance to a previous work in which CRISPR/Cas9‐mediated gene correction of Cockayne syndrome‐causing ERCC6 mutations in iPSCs further differentiated into MSCs (resulting from no or very low, respectively, to considerable CSB expression levels) has been shown to rescue the cells from senescence (Wang et al., 2020).

Moreover, the key role of ERCC6 up‐regulation in HDFs evading UVB‐mediated SIPS shown here is in accordance with a previous study reporting that CSB hinders DNA damage response (DDR)‐dependent senescence by binding to the p21 promoter and down‐regulating its transcription in a p53‐independent manner (Crochemore et al., 2019). However, when resistant HDFs were exposed to ionizing radiation, they were ultimately driven to premature senescence, maintaining ERCC6 expression and UVB tolerance at similar levels to those of untreated resistant cells and at significantly higher levels than those of young or IS cells. This result is in accordance with and reinforces our finding that resistant cells represent a pre‐existing subpopulation of HDFs, inherently overexpressing ERCC6 before their exposure to repeated UVB treatment, as revealed by our confocal microscopy analysis of CSB‐immunostained HDFs.

It is well accepted that cellular senescence is a potent anticancer mechanism; however, accumulation of senescent cells may support the growth, aggressiveness, and/or invasiveness of cancer cells (Coppé et al., 2008; Guan et al., 2017; Krtolica et al., 2001; Papadopoulou & Kletsas, 2011). We found that colonies formed when A431 epidermoid carcinoma cells were cultured on top of resistant HDFs' monolayers were bigger than those of young and smaller than those of IS cells, while tumors formed by A431 cells when co‐injected subcutaneously in the back of SCID mice were in‐between those formed when they were co‐injected with young or IS cells. Notably, resistant HDFs' molecular profile in terms of senescence‐related markers was also found to be in‐between young and senescent cells, occasionally resembling young (e.g., according to their p21^WAF1^, ICAM‐1, IL‐6, and MMP‐3 expression levels) or senescent (e.g., based on IL‐8, Col1a, fibronectin, α‐SMA, TGFβ1, IGFBP‐5, MMP‐1, and MMP‐13 gene expression) cells. In addition, MMP activity of resistant HDFs—which was assessed given that MMP secretion has been incriminated in tumor promotion by senescent human fibroblasts (Liu & Hornsby, 2007; Papadopoulou & Kletsas, 2011)—was found to be smaller than that of IS cells but remained significantly higher than that of young cells.

In conclusion, here we described for the first time the occurrence of a UVB‐resistant population, emerging after the treatment of HDFs with repeated non‐cytotoxic doses of UVB radiation and coexisting in mixed cultures with UVB‐induced senescent HDFs. UVB‐resistant HDFs were characterized by overexpression of the CSB protein, revealed to be a main UVB‐protective molecule and the key mechanism conferring resistance toward UVB‐mediated SIPS. In addition, UVB‐resistant HDFs remained normal and formed a distinct population in‐between young and the well‐characterized ionizing radiation‐induced senescent cells, but still retaining numerous tissue‐impairing characteristics of the SASP, enhancing tumor promotion. Thus, although they temporarily evade senescence, resistant HDFs may still negatively affect skin homeostasis. Given that these resistant cells constitute a UVB‐induced phenotype characterized by undesirable traits that subserve skin photo‐damage and photoaging, their presence should be also taken into account in any research strategy for the discovery of novel photo‐protective compounds, as well as in the design of any targeted and efficient approach to prevent, delay, or cure skin deterioration.

## AUTHOR CONTRIBUTIONS

DK conceptualized the project. AF, MTA, HP, and EM performed the experiments. AF and EM were responsible for visualization. DK acquired funding. DK supervised the project. EM and DK wrote the original draft of the manuscript. All authors reviewed the manuscript.

## CONFLICT OF INTEREST STATEMENT

None declared.

## Supporting information


Figures S1–S6.

Tables S1–S3.

## Data Availability

The data that supports the findings of this study are available in the supplementary material of this article. RNA‐seq data can be obtained from the GEO database under accession number GSE268564.
