# Supplementary material for: A subset of human dermal fibroblasts overexpressing Cockayne syndrome group B protein resist UVB radiation‐mediated premature senescence
Source: Aging Cell. 2024 Dec 19;24(3):e14422. doi: 10.1111/acel.14422 (PMC11896172; doi:10.1111/acel.14422)
Supplement: Supplementary file 1 — Figures S1–S6. [file ACEL-24-e14422-s002.docx]

**Supplementary Figure S1.**

**
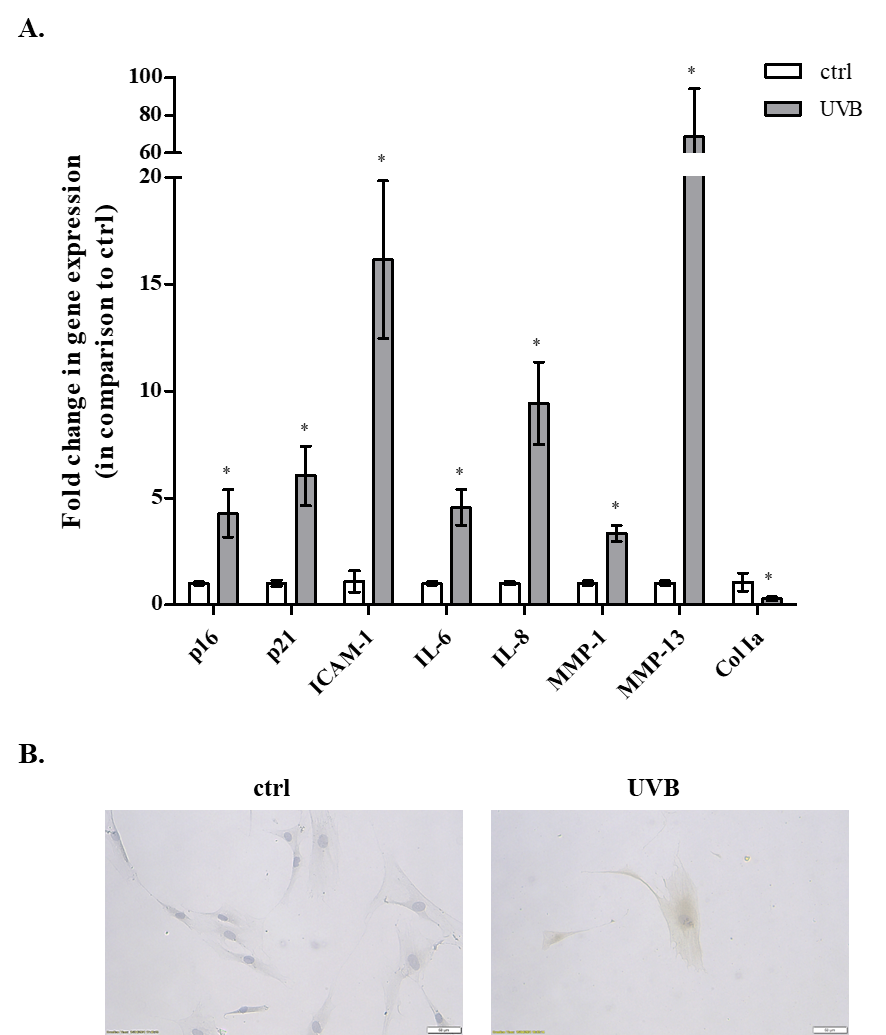
**

**Figure S1**

Cultures of human dermal fibroblasts subjected to a 10x35 mJ/cm^2^ UVB irradiation scheme 3 days post-UVB irradiation express as a whole several established biomarkers of senescence. A. Total RNA was extracted from control (ctrl) and UVB-treated (UVB) cells and was then used for RT-qPCR analysis using specific primers for the designated genes. Glyceraldehyde-3-phosphate dehydrogenase (GAPDH) was used as the reference gene. Numerical values are the means ± standard deviations of at least two independent experiments conducted in duplicates. Asterisks denote statistically significant differences in comparison to ctrl for every gene tested (Student’s t-test, p < 0.05). B. Untreated (ctrl) and exposed to a 10x35 mJ/cm^2^ UVB irradiation scheme (UVB) human dermal fibroblasts were allowed to attach onto glass coverslips for 3 days before their fixation and the staining of lipofuscin-containing cells with the SenTraGor reagent. Representative microscopic pictures are shown here. Scale bar: 50 μm.

**Supplementary Figure S2.**


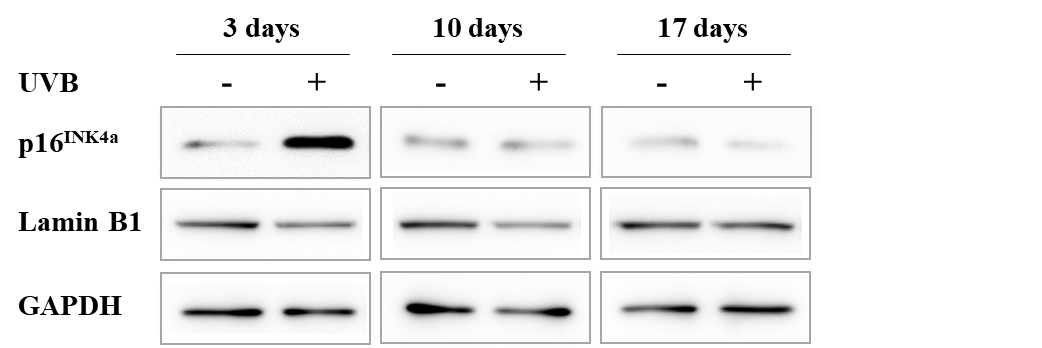


**Figure S2**

Human dermal fibroblasts repeatedly exposed to UVB irradiation show a progressive loss of the typical senescence markers that they initially express. Cells were subjected or not to repeated UVB radiation doses (10x35 mJ/cm^2^) before protein extraction 3, 10 and 17 days post-irradiation and western blot analysis for p16^INK4a^ and lamin B1. Western blot analysis for glyceraldehyde-3-phosphate dehydrogenase (GAPDH) was also performed to confirm equal loading. Representative blots are shown here.

***
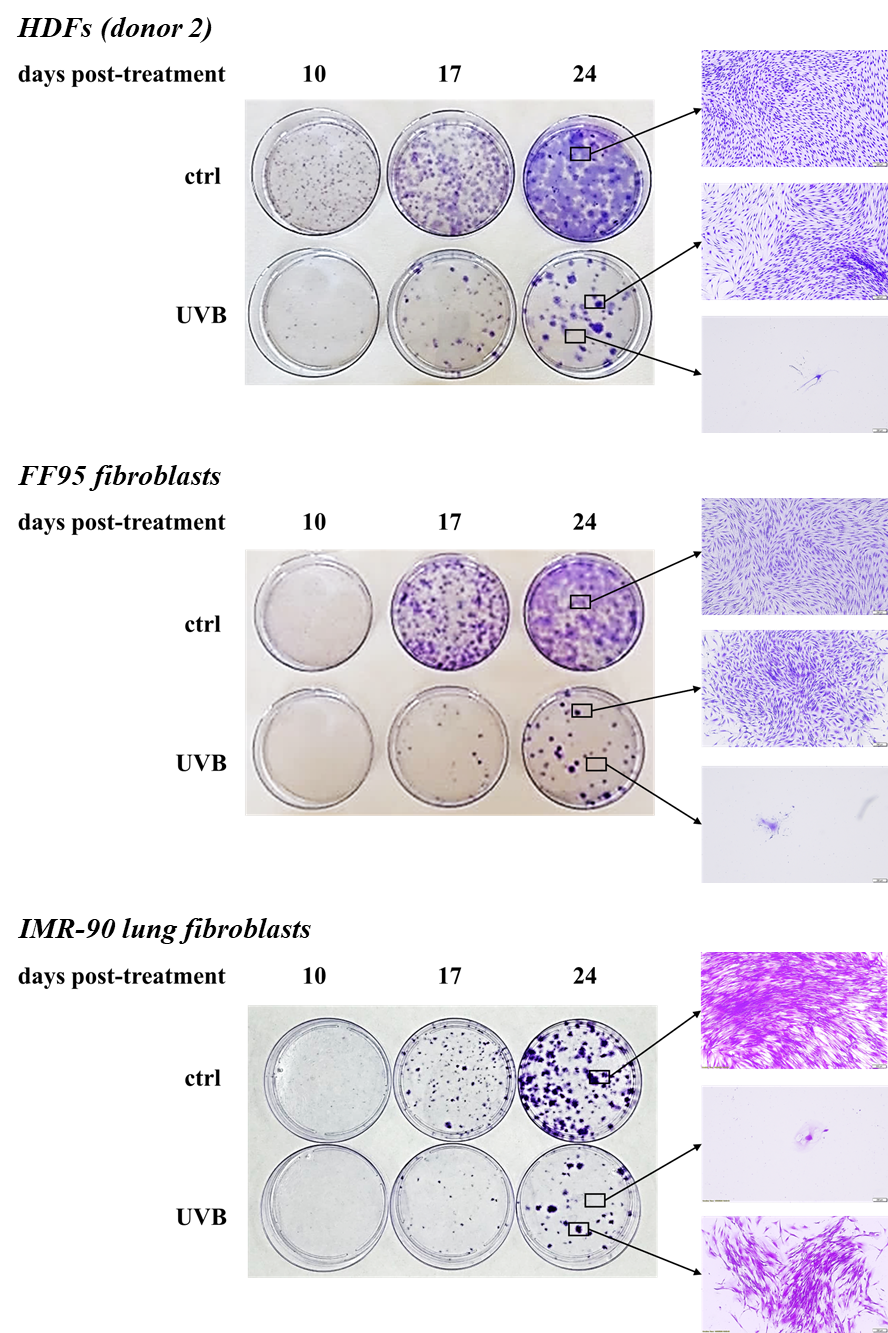
*Supplementary Figure S3.**

**Figure S3**

A subset of UVB-treated normal fibroblast cell strains regain their proliferative potential after an initial growth arrest. Untreated cells (ctrl) and cells exposed to their highest non-cytotoxic dose of UVB irradiation twice a day for five days (UVB) were very sparsely plated and left to grow onto the surface of petri dishes for 10, 17 and 24 days. After fixation, colonies formed were stained with crystal violet. HDFs: human dermal fibroblasts

**Supplementary Figure S4.**


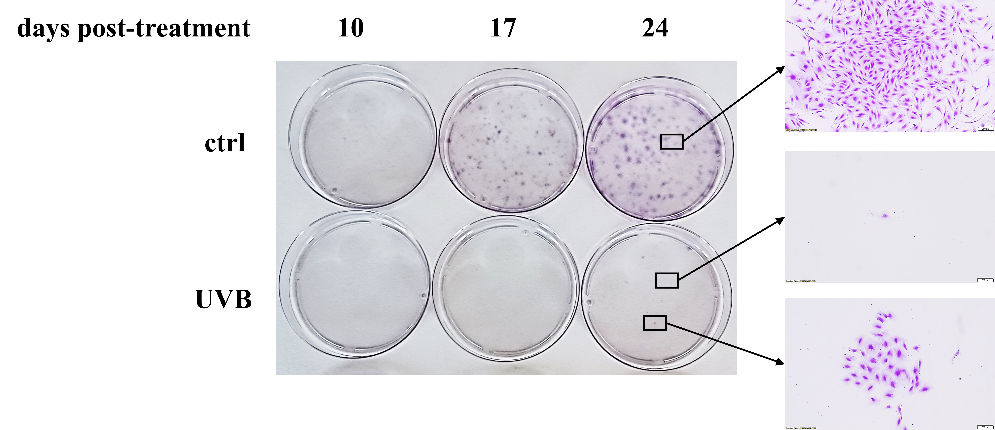


**Figure S4**

Dermal fibroblasts from a Cockayne syndrome patient carrying a mutation in the ERCC6 gene resulting in the loss of a functional CSB protein (GM00739) form a lower number of colonies compared to human dermal fibroblasts from normal donors. Untreated cells (ctrl) and cells exposed to their highest non-cytotoxic dose of UVB irradiation twice a day for five days (UVB) were very sparsely plated and left to grow onto the surface of petri dishes for 10, 17 and 24 days. After fixation, colonies formed were stained with crystal violet.

**Supplementary Figure S5.**


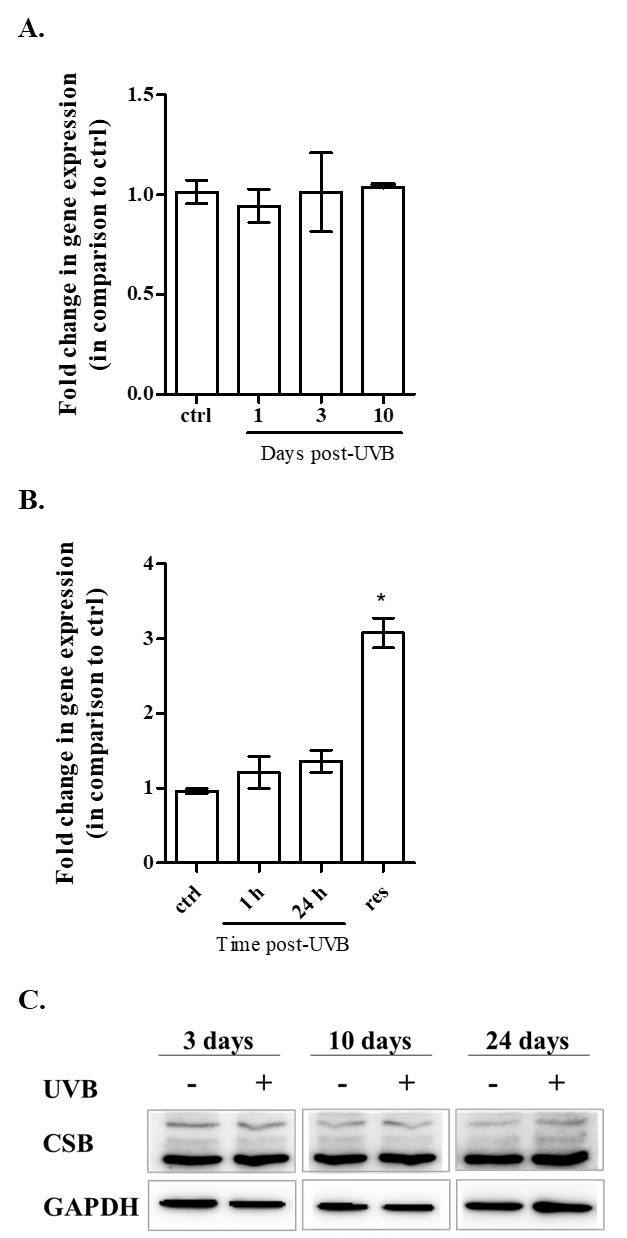


**Figure S5**

A single UVB radiation dose of 35 mJ/cm^2^ does not enhance ERCC6 gene expression, while ERCC6 mRNA levels and CSB protein expression rise only several days after the end of the repeated UVB radiation doses’ scheme (10x35 mJ/cm^2^), when resistant cells dominate in the culture. A. Human dermal fibroblasts were exposed to a single 35 mJ/cm^2^ dose of UVB irradiation before total RNA extraction at the designated time-points and RT-qPCR analysis for ERCC6. B. Human dermal fibroblasts were subjected to repeated UVB radiation doses (10x35 mJ/cm^2^) before total RNA extraction at the designated time-points up to 24 h, followed by RT-qPCR analysis for ERCC6. Resistant cells served as the positive control. C. Human dermal fibroblasts were subjected or not to repeated UVB radiation doses (10x35 mJ/cm^2^) before protein extraction 3, 10 and 24 days post-irradiation, followed by western blot analysis for CSB. Glyceraldehyde-3-phosphate dehydrogenase (GAPDH) served as the reference gene in RT-qPCR analysis and as the loading control in western blot analysis. Numerical values are the means ± standard deviations. Asterisk denotes statistical significance in comparison to control (ctrl) (Student’s t-test, p < 0.05). Representative blots are shown here.

**
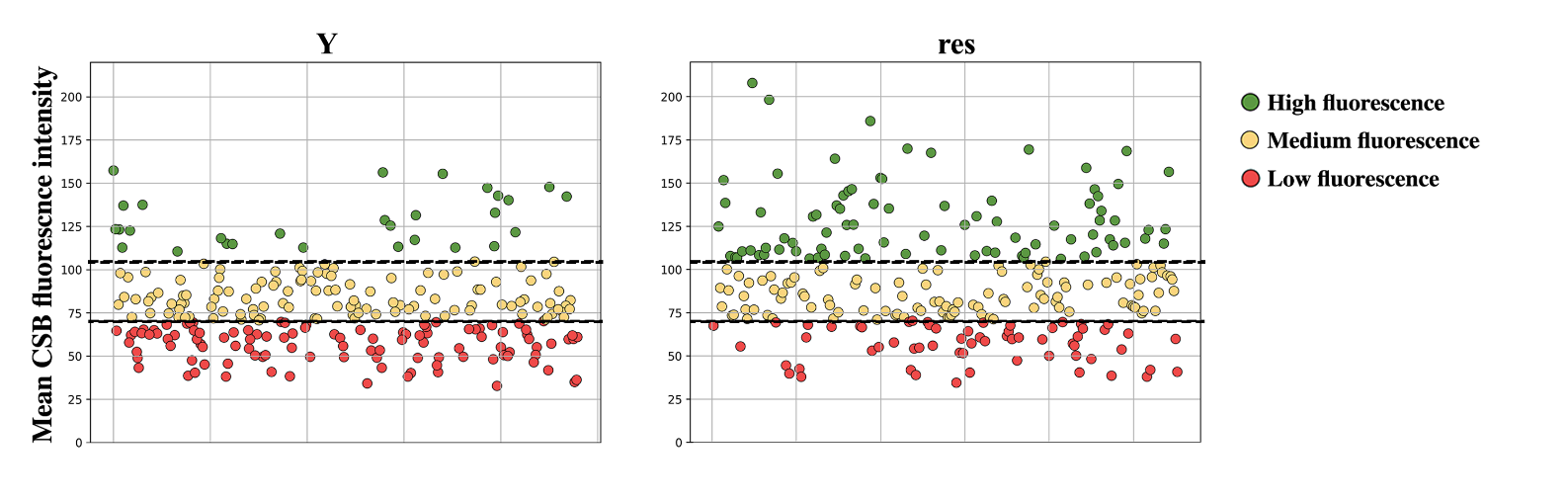
Supplementary Figure S6.**

**Figure S6**

Young untreated human dermal fibroblasts are divided in three subpopulations based on their CSB mean fluorescence intensity (corresponding to CSB protein expression) distribution across the nuclei (low, medium and high) and the high mean CSB fluorescence intensity cluster is amplified in resistant cells. Quantification was performed in multichannel Z-stack images captured using confocal laser scanning microscopy. Scatter plots illustrate the classification of young and resistant human dermal fibroblasts’ nuclei into distinct subgroups according to their mean CSB fluorescence intensity, based on the K-means clustering of young cells. K-means clustering for young human dermal fibroblasts was performed using the *scikit-learn* library for Python. *Mean CSB fluorescence intensity* feature served as the input variable for clustering with an optimal number of clusters *K=3*, as determined based on exploratory analysis and visual inspection of the data. Nuclei in the scatter plots are colored by their assigned cluster. Statistically significant differences were revealed between all pairwise comparisons of the fluorescence level’ s clusters in young cells (ANOVA, Tukey’s test, p < 0.05). Representative scatter plots for young and resistant populations (n=250) are depicted here. Dashed lines define the limits of the clusters. Y: young, res: resistant.
